# Health-related quality of life among older adults following acute hospitalization: longitudinal analysis of a randomized controlled trial

**DOI:** 10.1007/s11136-024-03689-x

**Published:** 2024-06-17

**Authors:** Eirin Guldsten Robinson, Hanna Gyllensten, Anne Gerd Granas, Kjell H. Halvorsen, Beate Hennie Garcia

**Affiliations:** 1 Department of Pharmacy, University of Oslo, Oslo, Norway; 2 Institute of Health and Care Sciences, Sahlgrenska Academy, University of Gothenburg, Gothenburg, Sweden; 3 Department of Pharmacy, Faculty of Health Sciences, UiT the Arctic University of Norway, Tromsø, Norway; 4 Hospital Pharmacy of North Norway Trust, Tromsø, Norway

**Keywords:** Health-related quality of life, Older adults, Integrated medication management, Medication optimization intervention, Medication review

## Abstract

**Purpose:**

To describe the longitudinal change of health-related quality of life (HRQoL) over 12 months from acute hospitalization in older adults ≥ 70 years (IMMENSE study), and associated factors, to investigate how a medication optimization intervention influenced this change.

**Methods:**

The EQ-5D-3L was used at discharge and 1, 6 and 12 months after discharge during a randomized controlled trial including 285 participants. Multilevel logistic (EQ-5D-3L dimensions) and mixed model regression (EQ-5D-3L index scores, EQ-VAS) were used to explore the longitudinal change with/without the intervention, and associations with medications, comorbidities, and socioeconomic variables. Subgroup analyses were performed for non-long and long stayers with hospitalizations < or ≥ 14 days.

**Results:**

EQ-5D-3L index scores significantly declined after 12 months (β −0.06 [95% confidence interval (CI:) −0.10–−0.02], *p* = *0.003*). Non-long stayers showed significant improvement 1 month from discharge (β 0.05 [0.00–0.09], *p* = *0.040*). The number of medications and receiving home-care services were the main factors associated with reduced HRQoL. Being home-dwelling was the main factor associated with higher HRQoL. Non-long stayers of the intervention group reported significantly higher EQ-VAS than the control group (β 4.02 [0.11–7.93], *p* = *0.044*).

**Conclusion:**

We observed no significant difference in the longitudinal change in HRQoL between the two IMMENSE study groups over 12 months after hospitalization. However, the non-long stayer subgroup analysis indicates that the intervention may have had a long-term effect on HRQoL in some of intervention patients. The number of medications and the ability to live and care for oneself should be taken into consideration when planning future patient care and health-care services.

**Trial registration:**

The trial was registered in clinicaltrials.gov on 28/06/2016 before enrolment started (NCT02816086).

**Supplementary Information:**

The online version contains supplementary material available at 10.1007/s11136-024-03689-x.

## Plain English summary

In this study we have described how the health-related quality of life in older adults developed in the year following an acute hospital admission. We explored the association between health-related quality of life and age, gender, living status, diseases and the use of multiple medications. Additionally, we investigated how an intervention to optimize medication use influenced the health-related quality of life. We report our results for patients in a subgroup with prolonged hospital stays (assumed to be less frail), compared to a subgroup without prolonged hospital stays (assumed to be frailer).

In the year following acute hospitalization, the patients without prolonged stays reported improved health-related quality of life 1 month after discharge, and their health-related quality of life did not decline in the follow-up period. Patients with prolonged hospital stays had a gradually declining health-related quality of life. Being home-dwelling was associated with better health-related quality of life. Using multiple medications and needing home-care services were associated with poorer health-related quality of life. Subjectively reported health-related quality of life was higher among patients receiving the intervention to optimize medication use compared to those receiving standard care in the patients without prolonged hospital stays.

## Background

Older adults are susceptible to medication-related harm due to age-related changes in pharmacodynamics and pharmacokinetics. With increasing age, multimorbidity and subsequent polypharmacy becomes more frequent in the population and are associated with deteriorating health-related quality of life (HRQoL) [1–6]. In addition, potentially inappropriate prescribing has been linked to adverse drug events (ADE) and adverse drug reactions (ADR), further compromising HRQoL among older adults [7–9].

Efforts to individualize and optimize prescribing through medication reviews have been acknowledged to enhance survival [10] and reduce healthcare use [11], among older adults. A recently published Cochrane review found that the number needed to treat for such interventions was 29 to prevent one hospital admission [12]. However, the authors highlighted a lack of published HRQoL data.

HRQoL has been recognized as a core outcome in studies aiming to improve medication appropriateness in older adults with polypharmacy [13, 14], i.e., an outcome that should always be included. Consequently, the IMMENSE study, a randomized controlled trial of a medication optimization intervention involving medication reviews in older adults after an acute hospital admission, included evaluation of the intervention effect on HRQoL [15]. The hypothesis was that optimized medication use has the potential to improve or prevent deterioration in HRQoL. Moreover, covariates collected in the study provides an opportunity to study additional factors associated with HRQoL in the study population.

The aim of this study was to describe the longitudinal change of health-related quality of life (HRQoL) over 12 months from acute hospitalization in older adults ≥ 70 years (IMMENSE study), and associated factors, to investigate how a medication optimization intervention influenced this change.

## Methods

### Study participants and design

This is an analysis of a secondary endpoint of the IMMENSE study, the change in HRQoL over 12 months from hospital discharge. The analysis employs HRQoL-data collected during the study conduct and health register data for the included patients. Previous publications from the study include the study protocol [15], an effectiveness evaluation which did not include the secondary endpoint of HRQoL [16], an intervention fidelity analysis [17] and a cost-utility analysis, which uses quality-adjusted life years derived from the HRQoL data, but without detailed analysis of its dimensions [18]. The study was conducted according to Good Clinical practice and the Declaration of Helsinki and reported according to the Consolidated Standards of Reporting Trials [15, 16].

Patients were randomized to the intervention or control group (1:1). The intervention group received the medication optimization intervention in addition to standard care, while the control group received standard care. Study participants were recruited at two internal medicine wards: one geriatric ward (Ward 1) and one general medicine ward (Ward 2) from September 2016 to December 2019. According to the study protocol patients for whom next of kin provided informed consent were excluded from HRQoL measurements [15]. Thus, in the current analysis we have included the 285 patients providing informed consent, out of the 480 IMMENSE study participants (intervention group n = 148 and control group n = 137).

During the IMMENSE study conduct it became evident that some patients remained in the hospital despite being ready for discharge. These prolonged stays were either due to no available beds in nursing homes or a lack of capacity in home-care services. Our data do not differentiate between patients who experienced extended hospital stays for medical reasons and those who were affected by this capacity problem. We therefore scrutinized the distribution of extended stays between the study groups before unblinding the group allocation, applying a cut-off of ≥ 14 days (post hoc), as this was twice the length of a mean hospital stay in our data material. As prolonged hospital stays are likely to be associated with the HRQoL of the participants, we report all results for the full population and in subgroups of *long stayers* (patients with at least one index stay or readmission ≥ 14 days), and *non-long stayers* (patients with no such extended hospitalizations).

### The intervention

The intervention comprised five steps aiming to optimize medication therapy, i.e., (i) medication reconciliation, (ii) medication review, (iii) patient counselling, (iv) comprehensible dissemination of medication list with explanations in the discharge summary, and (v) post-discharge phone call to the patient’s general practitioner or nursing home physician/nurse. The first four steps were completed during the index hospital stay. The fifth step was completed shortly after discharge, aiming to improve communication of recommendations across care levels.

### Health-related quality of life measurements

The EQ-5D-3L was used to collect HRQoL data at discharge, and 1, 6, and 12 months after discharge. The instrument comprises the EQ-5D-3L descriptive system and the EQ-VAS. The EQ-5D-3L descriptive system comprises five dimensions (mobility, self-care, usual activities, pain/discomfort, and anxiety/depression) with three possible response levels (no problems, moderate problems, or severe problems). The EQ-VAS records the patient’s subjectively reported health on a scale from 0 to 100 (100 indicating perfect health). A trained study nurse blinded to group allocation conducted the data collection by interviewing the patients. In Ward 1 this was done in person at the time of discharge and through telephone interviews for the subsequent times. For patients in Ward 2 all the EQ-5D-3L data were collected through telephone interviews.

We derived EQ-5D-3L index scores, translating the EQ-5D-3L responses for each collection time to a combined score between one for perfect health, zero for death, or below zero for conditions worse than death [19]. As no Norwegian tariff is available, Norwegian authorities recommend using the United Kingdom time-trade-off societal value set [20]. Patients who died during the follow-up were assigned a utility value of 0 at the collection times following their death.

### Sociodemographic and comorbidity data

Age, sex, marital status, living arrangements, level of education, the use of home-care services, the use of multidose adherence aid and medications in use were collected at study baseline. Comorbidities recorded at admission and discharge from the index admission were used to calculate the Charlson Comorbidity Index (CCI). The CCI was used as an alternative operationalization of comorbidities in the analyses, along with the individual comorbidities. Date of death was obtained from the Norwegian Cause of Death Registry, while hospital readmissions and length of stay were obtained from the Norwegian Patient Registry and the hospital’s cost per patient register.

### Statistical analysis

Patient characteristics at baseline were described as means with standard deviations (SD) for continuous variable, and as frequencies and percentages for categorical variables. Descriptive statistics for responses to the EQ-5D-3L dimensions were reported as frequencies and percentages at discharge from the index hospital stay, and at 1 month, 6 months and 12 months after discharge.

Factors associated with the EQ-5D-3L dimensions were identified using multilevel logistic regression models for each dimension (dichotomized: no problem versus some problems, i.e., moderate or extreme problem) as dependent variables following a step-by-step purposeful selection of variables approach [21]. First, univariable logistic regression analyses were conducted for all covariates. Multivariable models were subsequently built, selecting all independent variables with *p-values* < *0.25* from the univariable analyses. Finally, variables with a *p-value* > *0.05* or yielding a change in coefficients < 20%) were removed in steps, testing for model fit using a likelihood ratio test, to create the final model [21]. Individual comorbidities and CCI were tested in separate sets of models as a sensitivity analysis of how comorbidity is operationalized.

Factors associated with EQ-5D-3L index scores and EQ-VAS were identified as dependent variables in multivariable linear mixed-effects models accounting for the longitudinal nature of the HRQoL data [22]. Independent variables were selected following the same purposeful selection approach as for the logistic regression models. Clustering on the patient level and study ward level was explored in all regression analyses. Missing EQ-5D-3L index scores for any collection times were 9.1% and were assumed to be missing at random. Mixed-model regression was used to model both the multi-level structure of the data in terms of repeated measures for each patient, and missing timepoints after imputation of zero for deceased patients [23–25]. Results from the logistic and mixed model regressions are reported as odds ratio (OR) and beta coefficient (β), respectively.

All statistical analyses were carried out using Stata 17 [26].

## Results

### Characteristics of the study population

The study population had a mean age of 82.5 years with almost 70% of the population ≥ 80 years old. Before study inclusion, more than 90% of the study population were home-dwelling, and almost 60% lived alone. After the index admission, 75.4% of the population were discharged to their homes. The mean total number of medications at baseline was 9.1, with 81% of the population using ≥ 5 medications. After discharge, the mean total number of medications was 10.3, with 89% of the population using ≥ 5 medications. The most prevalent comorbidities were hypertension (51%), asthma or COPD (29%), and atrial fibrillation (28%), with a mean CCI score of 2.3 (SD 1.9). A total of 9.1% of the study population died during the 12-month follow-up period (Table 1).

**Table 1:** Characteristics of the non-long stayers (n = 222), long stayers (n = 63) and total population (N = 285)

	Full population			Non-long stayers			Long stayers		
	Intervention group (N = 148)	Control group (N = 137)	Total (N = 285)	Intervention group (N = 106)	Control group (N = 116)	Total (N = 222)	Intervention group (N = 42)	Control group (N = 21)	Total (N = 63)
Age, years									
Mean (SD)	82.8 (6.0)	82.1 (5.9)	82.5 (6.0)	82.4 (6.1)	81.9 (5.9)	82.2 (6.0)	83.9 (5.9)	82.9 (6.1)	83.5 (5.9)
70–74 years, n (%)^a^	16 (10.8)	21 (15.3)	37 (13.0)	12 (11.3)	19 (16.4)	31 (14.0)	4 (9.5)	2 (9.5)	6 (9.5)
75–79 years, n (%)^a^	27 (18.2)	24 (17.5)	51 (17.9)	20 (18.9)	19 (16.4)	39 (17.6)	7 (16.7)	5 (23.8)	12 (19.1)
80–84 years, n (%)^a^	44 (29.7)	42 (30.7)	86 (30.2)	32 (30.2)	39 (33.6)	71 (32.0)	12 (28.6)	3 (14.3)	15 (23.8)
≥ 85 years, n (%)^a^	61 (41.2)	50 (36.5)	111 (39.0)	42 (39.6)	39 (33.6)	81 (36.5)	19 (45.2)	11 (52.4)	30 (47.6)
Sex, n (%)									
Female	74 (54.0)	98 (66.2)	172 (60.4)	69 (65.1)	65 (56.0)	134 (60.4)	29 (69.1)	9 (42.9)	38 (60.3)
Level of education, n (%)									
Low (< 12 years)	75 (52.8)	73 (54.1)	148 (53.4)	52 (49.1)	64 (55.2)	116 (52.3)	23 (54.8)	9 (42.9)	32 (50.8)
High (> 12 years)	67 (47.2)	62 (45.9)	129 (46.6)	50 (47.2)	50 (43.1)	100 (45.1)	17 (40.5)	12 (57.1)	29 (46.0)
Missing	6 (4.1)	2 (1.5)	8 (2.8)	4 (3.8)	2 (1.7)	6 (2.7)	2 (4.8)	0 (0.0)	2 (3.2)
Living status, n (%)									
Home-dwelling before included	137 (92.6)	129 (94.2)	266 (93.3)	100 (94.3)	111 (95.7)	211 (95.1)	37 (88.1)	18 (85.7)	55 (87.3)
Living alone before included	86 (58.5)	82 (59.9)	168 (59.2)	59 (56.2)	69 (59.5)	128 (57.9)	27 (64.3)	13 (61.9)	40 (63.5)
Discharged home	113 (76.4)	101 (74.3)	214 (75.4)	91 (85.9)	91 (78.5)	182 (82.0)	22 (52.4)	10 (50.0)	32 (51.6)
Need for assistance, n (%)									
Home-care services	75 (50.7)	77 (56.2)	152 (53.3)	48 (45.3)	63 (54.3)	111 (50.0)	27 (64.3)	14 (66.7)	41 (65.1)
Multidose adherence aid	43 (29.0)	48 (35.0)	91 (31.9)	28 (25.5)	38 (32.8)	65 (29.3)	16 (38.1)	10 (47.6)	26 (41.3)
Handling own medications	77 (52.0)	64 (46.7)	141 (49.5)	59 (55.7)	58 (50.0)	117 (52.7)	18 (42.9)	6 (28.6)	24 (38.1)
Medication use, admission, mean (SD)									
Number of medications regular use	6.6 (4.0)	7.4 (4.0)	7.0 (4.0)	6.8 (4.1)	7.2 (4.0)	7.0 (4.0)	6.4 (3.9)	8.5 (3.9)	7.1 (4.0)
Number of medications total	8.7 (5.1)	9.6 (5.4)	9.1 (5.2)	8.8 (5.1)	9.4 (5.4)	9.1 (5.3)	8.5 (5.1)	10.5 (5.1)	9.1 (5.2)
Medications total < 5, n (%)^a^	31 (21.0)	23 (16.8)	54 (19.0)	20 (18.9)	22 (19.0)	42 (18.9)	11 (26.2)	1 (4.8)	12 (19.1)
Medications total ≥ 5, n (%)^a^	117 (79.1)	114 (83.2)	231 (81.1)	86 (81.1)	94 (81.0)	180 (81.1)	31 (73.8)	20 (95.2)	51 (81.0)
Medication use, discharge, mean (SD)									
Number of medications regular use	7.0 (3.6)	7.6 (3.8)	7.3 (3.7)	6.9 (3.5)	7.5 (3.9)	7.2 (3.7)	7.3 (3.8)	8.1 (3.4)	7.6 (3.6)
Number of medications total	10.3 (5.0)	10.4 (5.5)	10.3 (5.2)	10.2 (5.0)	10.3 (5.6)	10.2 (5.3)	10.8 (5.0)	10.7 (4.5)	10.7 (4.8)
Medications total < 5, n (%)^a^	16 (10.8)	16 (11.7)	32 (11.2)	12 (11.3)	15 (12.9)	27 (12.2)	4 (9.5)	1 (4.8)	5 (7.9)
Medications total ≥ 5, n (%)^a^	132 (89.2)	121 (88.3)	253 (88.8)	94 (88.7)	101 (87.1)	195 (87.8)	38 (90.5)	20 (95.2)	58 (92.1)
Charlson Comorbidity Index, mean (SD)	2.1 (1.8)	2.5 (2.0)	2.3 (1.9)	2.1 (1.8)	2.4 (2.0)	2.3 (1.9)	2.2 (1.7)	3.0 (1.7)	2.5 (1.7)
Comorbidities in admission notes, n (%)									
Hypertension	75 (51.7)	69 (50.4)	144 (50.5)	54 (50.9)	58 (50.0)	110 (49.6)	21 (50.0)	11 (52.4)	32 (50.8)
Asthma or COPD	44 (29.7)	38 (27.7)	82 (28.8)	34 (32.1)	33 (28.5)	67 (30.2)	10 (23.8)	5 (23.8)	15 (23.8)
Atrial fibrillation	37 (25.0)	42 (30.7)	79 (27.7)	25 (23.6)	36 (31.0)	61 (27.5)	12 (28.6)	6 (28.6)	18 (28.6)
Diabetes	28 (18.9)	31 (22.6)	59 (20.7)	21 (19.8)	25 (21.6)	46 (20.7)	7 (16.7)	6 (28.6)	13 (20.6)
Heart failure	24 (16.2)	21 (15.3)	45 (15.8)	14 (13.2)	17 (14.7)	31 (14.0)	10 (23.8)	4 (19.1)	14 (22.2)
Renal failure	24 (16.2)	21 (15.3)	45 (15.8)	14 (13.2)	16 (13.8)	30 (13.5)	4 (9.5)	0 (0.0)	4 (6.4)
Anxiety/depression	17 (11.5)	11 (8.0)	28 (9.8)	11 (10.4)	10 (8.6)	21 (9.5)	10 (23.8)	5 (23.8)	15 (23.8)
Dementia	7 (4.7)	4 (2.9)	11 (3.9)	3 (2.8)	4 (3.5)	7 (3.2)	4 (9.5)	0 (0.0)	4 (6.4)
Died during the study period, n (%)	14 (9.5)	12 (8.8)	26 (9.1)	7 (6.6)	10 (8.6)	17 (7.7)	7 (16.7)	2 (9.5)	9 (14.3)
Study ward, n (%)									
Ward 1	117 (79.1)	103 (75.2)	220 (77.2)	80 (75.5)	85 (73.3)	165 (74.3)	37 (88.1)	18 (85.7)	55 (87.3)
Ward 2	31 (21.0)	34 (24.8)	65 (22.8)	26 (24.5)	31 (26.7)	57 (25.7)	5 (11.9)	3 (14.3)	8 (12.8)

^a^Percentages were rounded

*COPD* Chronic Obstructive Pulmonary Disease; *SD* standard deviation; *CI* confidence interval

### EQ-5D-3L dimensions

No statistically significant differences in the EQ-5D-3L dimensions were identified between the study groups (Table 3).

At discharge, 91.2% of the patients reported at least one moderate or extreme problem in at least one of the dimensions in both the intervention group (I) and control group (C), respectively (Table 2). The proportion of missing responses was low at discharge (I: 1.4% and C: 0.0%), but gradually increased throughout the study period (I: 17.0% and C: 15.2% at 12 months). The most frequently reported problems (moderate or extreme) at discharge were Mobility (I: 75.0% and C: 76.6%), Usual Activities (I: 71.6% and C: 72.3%) and Pain/discomfort (I: 66.2% and C: 62.0%). Extreme problems at discharge were most frequently reported for Usual activities (I: 27.4% and C: 32.9%) and Pain/discomfort (I: 12.3% and C: 13.4%) dimensions. The dimensions with frequent problems remained high throughout the study period. Results for the non-long and long stayers can be found in Supplementary Tables S1 and S2, respectively. Reported problems in Self-care were significantly reduced 6 months after discharge for the full population (odds ratio (OR) [with 95% confidence interval]: 0.39 [0.23–0.67], *p* = *0.001*) (Table 3). This effect was also evident among the non-long stayers (0.31 [0.16–0.59], *p* < *0.001*) (Table S3), but not among the long stayers (Table S4).

**Table 2:** Problems in the EQ-5D-3L dimensions, EQ-5D-3L index score and EQ-VAS in the full population (n = 285)

	Discharge N = 285		1 month N = 277		6 months N = 268		12 months N = 259	
	Intervention group n = 148	Control group n = 137	Intervention group n = 145	Control group n = 132	Intervention group n = 139	Control group n = 129	Intervention group n = 134	Control group n = 125
Mobility, n (%)								
No problems	35 (24.0)	32 (23.4)	41 (28.3)	29 (22.0)	35 (25.2)	23 (17.8)	30 (22.2)	18 (14.4)
Moderate problems	101 (69.2)	92 (67.2)	86 (59.3)	86 (65.2)	83 (59.7)	85 (65.9)	72 (53.3)	79 (63.2)
Extreme problems	10 (6.9)	13 (9.5)	8 (5.5)	7 (5.3)	4 (2.9)	8 (6.2)	10 (7.4)	9 (7.2)
Reporting some problems^a^	111 (75.0)	105 (76.6)	94 (64.8)	93 (70.5)	87 (62.6)	93 (72.1)	82 (60.7)	88 (70.4)
Missing	2 (1.4)	0 (0)	10 (6.9)	10 (7.6)	17 (12.2)	13 (10.1)	23 (17.0)	19 (15.2)
Self-care, n (%)								
No problems	93 (63.7)	83 (60.6)	87 (60.0)	85 (64.4)	95 (68.4)	84 (65.1)	77 (57.0)	78 (62.4)
Moderate problems	43 (29.5)	36 (26.3)	42 (29.0)	29 (22.0)	22 (15.8)	24 (18.6)	29 (21.5)	19 (15.2)
Extreme problems	10 (6.9)	18 (13.1)	6 (4.1)	8 (6.1)	5 (3.6)	7 (5.4)	6 (4.4)	9 (7.2)
Reporting some problems^a^	53 (35.8)	54 (39.4)	48 (33.1)	37 (28.0)	27 (19.4)	31 (24.0)	35 (25.9)	28 (22.4)
Missing	2 (1.4)	0 (0)	10 (6.9)	10 (7.6)	17 (12.2)	14 (10.8)	23 (17.0)	19 (15.2)
Usual activities, n (%)								
No problems	40 (27.4)	38 (27.7)	48 (33.1)	30 (22.7)	38 (27.3)	31 (24.0)	38 (28.15)	29 (23.2)
Moderate problems	66 (45.2)	54 (39.4)	57 (39.3)	61 (46.2)	61 (43.9)	59 (45.7)	51 (37.8)	52 (41.6)
Extreme problems	40 (27.4)	45 (32.9)	30 (20.7)	31 (23.5)	23 (16.6)	24 (18.6)	23 (17.0)	25 (20.0)
Reporting some problems^a^	106 (71.6)	99 (72.3)	87 (60.0)	92 (69.7)	84 (60.4)	83 (64.3)	74 (54.8)	77 (61.6)
Missing	2 (1.4)	0 (0)	10 (6.9)	10 (7.6)	17 (12.2)	15 (11.6)	23 (17.0)	19 (15.2)
Pain/discomfort, n (%)								
No problems	48 (32.9)	52 (38.0)	43 (29.7)	48 (36.4)	45 (32.4)	34 (26.4)	32 (23.7)	29 (23.2)
Moderate problems	80 (54.8)	66 (48.2)	77 (53.1)	60 (45.5)	61 (43.9)	60 (46.5)	63 (46.7)	59 (47.2)
Extreme problems	18 (12.3)	19 (13.4)	15 (10.3)	14 (10.6)	16 (11.5)	20 (15.5)	17 (12.6)	18 (14.4)
Reporting some problems^a^	98 (66.2)	85 (62.0)	92 (63.5)	74 (56.1)	77 (55.4)	80 (62.0)	80 (59.3)	77 (61.6)
Missing	2 (1.4)	0 (0)	10 (6.9)	10 (7.6)	17 (12.2)	15 (11.6)	23 (17.0)	19 (15.2)
Anxiety/depression, n (%)								
No problems	96 (65.8)	76 (55.5)	86 (59.3)	78 (59.1)	75 (54.0)	64 (49.6)	70 (51.9)	65 (52.0)
Moderate problems	42 (28.8)	50 (36.5)	44 (30.3)	37 (28.0)	44 (31.7)	44 (34.1)	38 (28.2)	36 (28.8)
Extreme problems	8 (5.5)	11 (8.0)	5 (3.5)	6 (4.6)	3 (2.2)	6 (4.7)	4 (3.0)	4 (3.2)
Reporting some problems^a^	50 (33.8)	61 (44.5)	49 (33.8)	43 (32.6)	47 (33.8)	50 (38.8)	42 (31.1)	40 (32.0)
Missing	2 (1.4)	0 (0)	11 (8.3)	10 (7.6)	17 (12.2)	15 (11.6)	23 (17.0)	20 (16.0)
Any dimension, n (%)								
No problems	11 (7.4)	12 (8.8)	14 (9.7)	12 (9.1)	15 (10.8)	8 (6.2)	7 (5.2)	9 (7.2)
Moderate problems	79 (53.4)	64 (46.7)	78 (53.8)	68 (51.5)	69 (49.6)	70 (54.3)	68 (50.4)	61 (48.8)
Extreme problems	56 (37.8)	61 (44.5)	43 (29.7)	42 (31.8)	38 (27.3)	38 (29.5)	37 (27.4)	36 (28.8)
Reporting some problems^a^	135 (91.2)	125 (91.2)	121 (83.5)	110 (83.3)	107 (77.0)	108 (83.7)	105 (77.8)	97 (77.6)
Missing	2 (1.4)	0 (0)	10 (6.9)	10 (7.6)	17 (12.2)	13 (10.1)	23 (17.0)	19 (15.2)
EQ-5D-3L index score^b^	0.518	0.472	0.550	0.544	0.558	0.505	0.495	0.482
EQ-VAS score^b^	59.16	55.51	59.49	59.12	61.20	58.62	60.44	56.92

^a^Reporting some problems is the sum of moderate problems and extreme problems

^**b**^Unadjusted

**Table 3:** Results of multivariable multi-level logistic regression models of EQ-5D dimensions (no problem versus problem (moderate or extreme)) for the full population (n = 285)

	Mobility		Self-care		Usual activities		Pain/Discomfort		Anxiety/Depression	
	OR (95% CI)	p-value	OR (95% CI)	p-value	OR (95% CI)	p-value	OR (95% CI)	p-value	OR (95% CI)	p-value
Constant	0.02 (0.00–5.08)	0.167	0.12 (0.02–0.57)	0.008	0.01 (0.00–2.27)	0.099	0.54 (0.28–1.06)	0.167	45.09 (0.23–89.84)	0.159
Intervention group	0.72 (0.35–1.47)	0.365	1.25 (0.66–2.34)	0.437	0.86 (0.44–1.67)	0.656	1.18 (0.71–1.94)	0.525	0.69 (0.34–1.41)	0.312
Time (reference: discharge)										
1 month	0.75 (0.43–1.30)	0.306	0.78 (0.47–1.28)	0.318	0.82 (0.48–1.41)	0.467	0.99 (0.64–1.53)	0.960	0.81 (0.49–1.35)	0.433
6 months	1.12 (0.63–1.98)	0.707	**0.39 (0.23–0.67)**	**0.001**	1.02 (0.59–1.79)	0.081	1.13 (0.72–1.77)	0.593	1.36 (0.81–2.28)	0.242
12 months	1.73 (0.95–3.16)	0.075	0.78 (0.46–1.34)	0.366	1.08 (0.62–1.90)	0.784	1.69 (1.05–2.71)	0.031	1.07 (0.63–1.82)	0.797
Study ward 1	NS		**2.42 (1.04–5.66)**	**0.041**	**3.27 (1.43–7.47)**	**0.005**	NA		NS	
Age when included^a^	**1.08 (1.02–1.15)**	**0.010**	NS		**1.08 (1.02–1.14)**	**0.013**	NA		**0.93 (0.87–0.99)**	**0.025**
Sex Female	NA		NA		NS		**2.04 (1.22–3.40)**	**0.007**	**2.59 (1.24–5.41)**	**0.011**
Level of education > 12 years	NA		NA		NS		NA		NA	
Home-dwelling before included	**0.07 (0.01–0.46)**	**0.006**	**0.12 (0.03–0.42)**	**0.001**	**0.03 (0.00–0.22)**	**0.001**	NA		NS	
Living alone before included	NS		NS		NS		NA		NA	
Home-care services	**6.79 (2.95–15.64)**	** < 0.001**	**12.44 (5.71–27.10)**	** < 0.001**	**4.77 (2.18–10.43)**	** < 0.001**	NS		NS	
Number of medications total^a^	**1.11 (1.02–1.20)**	**0.012**	**1.10 (1.03–1.17)**	**0.003**	**1.24 (1.14–1.34)**	** < 0.001**	**1.12 (1.07–1.18)**	** < 0.001**	**1.09 (1.01–1.17)**	**0.023**
Handling own medications	NS		NS		NS		NA		**0.34 (0.15–0.76)**	**0.009**
Multidose adherence aid	NS		NS		NS		NS		NS	
Hypertension	NA		NA		NA		NA		NA	
Asthma or COPD	NA		NS		NS		NS		NA	
Atrial fibrillation	NS		NS		NS		NA		**2.58 (1.17–5.70)**	**0.019**
Diabetes	NS		NS		NS		NA		NS	
Heart failure	NS		NS		**0.36 (0.13–0.97)**	**0.043**	NA		NS	
Renal failure	NA		NA		NA		NA		NA	
Anxiety / depression	NA		NS		NS		NS		**19.94 (5.35–74.39)**	** < 0.001**
Dementia	NA		NS		NA		**0.15 (0.28–0.06)**	**0.007**	NA	
Charlson Comorbidity Index^b^	NS		NS		NS		NA		NA	

*COPD* Chronic Obstructive Pulmonary Disease; *OR* odds ratio; *CI* confidence interval; *NS* not significant in multivariate regression; *NA* not applicable based on univariate regression

^a^ Continuous varable

^b^ Tested separately from other comorbidities

Results in bold are statistically significant

The number of medications was significantly associated with reporting problems in each of the five dimensions: Mobility (1.11 [1.02–1.20], *p* = *0.012*), Self-care (1.10 [1.03–1.17], *p* = *0.003*), Usual activities (1.24 [1.14–1.34], p < 0.001) and Pain/Discomfort (1.12 [1.07–1.18], *p* < *0.001*) and Anxiety/Depression (1.09 [1.01–1.17], *p* = *0.023*), see Table 3. Being home-dwelling before inclusion was negatively associated with Mobility (0.07 [0.01–0.46], *p* = *0.006*), Self-care (0.12, [0.03–0.42], *p* = *0.001*), and Usual activities (0.03 [0.00–0.22], *p* = *0.001*). However, receiving home-care services was positively associated with reporting problems in the same dimensions: Mobility (6.79 [2.95–15.64], *p* < *0.001*), Self-care (12.44 [5.71–27.10], *p* < *0.001*) and Usual activities (4.77 [2.18–10.43], *p* < *0.001*). Being admitted to study Ward 1 was associated with problems in Self-care (2.42 [1.04–5.66], *p* = *0.041*) and Usual activities (3.27 [1.43–7.47], *p* = *0.005*) compared to study Ward 2. Age was associated with an 8% increase per year in odds of problems with both Mobility (1.08 [1.02–1.15], *p* = *0.001*) and Usual activities (1.08 [1.02–1.14], *p* = *0.013*) and a 7% reduction per year in odds of problems with Anxiety/Depression (0.93 [0.87–0.99], *p* = *0.025*). Being female was associated with a more than twofold higher odds of problems with both Pain/Discomfort (2.04 [1.22–3.40], *p* = *0.007*) and Anxiety/Depression (2.59 [1.24–5.41], *p* = *0.011*) in the multivariate logistic regression (Table 3).

In the multivariable regression model recorded anxiety or depression (19.94 [5.35–74.39], *p* < *0.001*) or atrial fibrillation (2.58 [1.17–5.70], *p* = *0.019*) in the admission- or discharge notes were associated with increased odds of Anxiety/depression. Dementia was associated with reduced odds of reporting problems with Pain/Discomfort (0.15 [0.28–0.06], *p* = *0.007*), and heart failure was associated with reduced odds of reporting problems in Usual activities (0.36 [0.13–0.97], *p* = *0.043*). Results for the non-long and long stayers and full population can be found in supplementary Tables S3 and S4 (multivariable regression) and S6 and S7 (univariable regression).

### EQ-5D-3L index scores

**Fig. 1:**
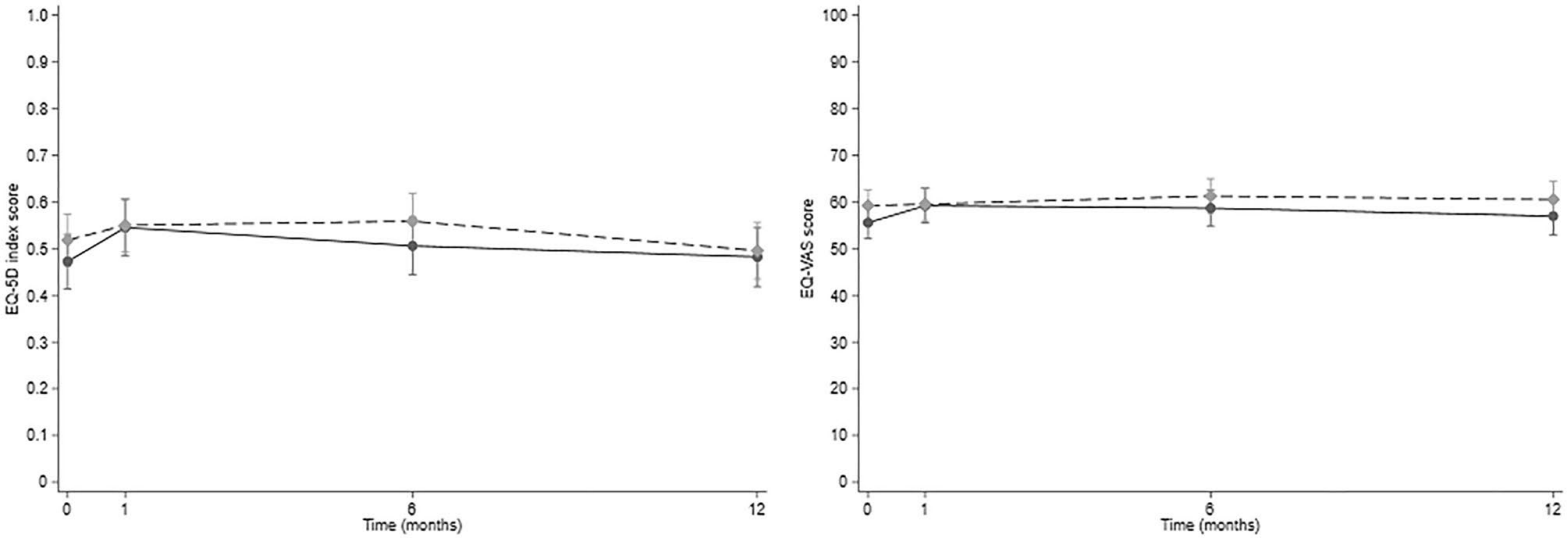
Mixed model marginal trajectories from discharge to 12 months for the EQ-5D-3L index score (left panel) and EQ-VAS (right panel) for the full population. The dashed line represents the marginal trajectories of the intervention group, and the solid line represents the marginal trajectories of the control group

The EQ-5D-3L index scores mixed model marginal trajectories for the study groups can be found in Fig. 1 (left panel). There were no statistically significant differences in the EQ-5D-3L index scores between the study groups.

In the full population, the EQ-5D-3L index score at discharge was 0.518 in the intervention group and 0.472 in the control group (Table 2). The EQ-5D-3L index scores significantly decreased 12 months after discharge in the full population (−0.06 [−0.10–−0.02], *p* = *0.003*) (Table 4) and in the long stayers (−0.17 [−0.27–−0.07], *p* = *0.001*) (Table S8). However, for the non-long stayers there was a significant improvement in the EQ-5D-3L index scores 1 month from discharge (0.05, 95%CI [0.00–0.09], *p* = *0.040*), and no significant decrease relative to discharge throughout the study period (Table S8).

**Table 4:** Results of univariable and final multivariable mixed model regressions of EQ-5D-3L index scores for the full population (n = 285)

	Univariable regression		Multivariable regression	
	β (95% CI)	p-value	β (95% CI)	p-value
Constant	0.51 (0.39–0.10)	< 0.001	0.80 (0.39–1.20)	< 0.001
Intervention group	0.04 (−0.03–0.10)	0.293	0.007 (–)	0.791
Time (reference: discharge)				
1 month	0.02 (−0.02–0.06)	0.396	0.02 (−0.02–0.06)	0.387
6 months	−0.01 (−0.06–0.03)	0.484	−0.02 (−0.06–0.03)	0.473
12 months	**−0.06 (−0.10–−0.02)**	**0.003**	**−0.06 (−0.10–−0.02)**	**0.003**
Study ward 1	**−0.17 (−0.25–−0.09)**	** < 0.001**	NS	
Age when included^a^	**−0.01 (−0.02–−0.01)**	** < 0.001**	−0.003 (0.155**–**)	0.229
Sex Female	**−0.06 (−0.13–0.01)**	**0.114**	NS	
Level of education > 12 years	0.02 (−0.05–0.09)	0.612	NA	
Home-dwelling before included	**0.20 (0.07–0.34)**	**0.003**	**0.22 (0.11–0.33)**	** < 0.001**
Living alone before included	−0.03 (−0.10–0.04)	0.380	NA	
Home-care services	**−0.26 (−0.32–−0.20)**	** < 0.001**	**−0.15 (−0.21–−0.08)**	** < 0.001**
Number of medications total^a^	**−0.03 (−0.03–−0.02)**	** < 0.001**	**−0.02 (−0.02–−0.01)**	** < 0.001**
Handling own medications	**0.25 (0.19–0.31)**	** < 0.001**	NS	NS
Multidose adherence aid	**−0.26 (−0.33–−0.19)**	** < 0.001**	**−0.11 (−0.18–−0.04)**	** < 0.001**
Hypertension	0.005 (-0.07**–**0.06)	0.894	NA	
Asthma or COPD	**−0.09 (−0.16–−0.01)**	**0.021**	NS	
Atrial fibrillation	−0.04 (−0.11–0.04)	0.318	NA	
Diabetes	**−0.09 (−0.17–−0.00)**	**0.040**	NS	
Heart failure	**−0.08 (−0.18–0.01)**	**0.086**	NS	
Renal failure	−0.02 (−0.12**–**0.07)	0.630	NA	
Anxiety / depression	**−0.15 (−0.27–−0.39)**	**0.009**	NS	
Dementia	**−0.17 (−0.34–0.01)**	**0.059**	NS	
Charlson Comorbidity Index^b^	**−0.03 (−0.05–−0.02)**	** < 0.001**	NS	

*COPD* Chronic Obstructive Pulmonary Disease; *CI* confidence interval; *β* regression coefficient; *NS* not significant in multivariate regression; *NA* not applicable based on univariate regression

^a^ Continuous varable

^b^ Tested in a separate model from other comorbidities

Results in bold are statistically significant

The number of medications (per additional medication) (−0.02 [−0.02–−0.01], *p* < *0.001*), receiving home-care services (yes/no) (−0.15 [−0.23–−0.07], *p* < *0.001*), and using multidose adherence aid (yes/no) (−0.11 [−0.18–−0.04], *p* < *0.001*) were associated with reduced EQ-5D-3L index scores in the final multivariable model. Being home-dwelling before admission was associated with higher EQ-5D-3L index scores (0.22 [0.11–0.33], *p* < *0.001*). In the univariable models, sex, age and several of the comorbidities were significantly associated with reduced EQ-5D-3L index scores, however, in the multivariable model none of the comorbidities were significant (Table 4).

### EQ-VAS

The EQ-VAS mixed model marginal trajectories for the study groups can be found in Fig. 1 (right panel). The non-long stayers of the intervention group reported a significantly improved EQ-VAS compared to the control group (β 4.02 95%CI [0.11–7.93], *p* = *0.044*) (Table S9). However, this effect was not evident in the full population or the long stayers.

In the full population the EQ-VAS score was 59.16 in the intervention group and 55.51 in the control group (Table 2). There were no significant longitudinal changes in EQ-VAS from discharge in the full population or the subgroups (Tables 5 and S9, respectively).

**Table 5:** Results of univariable and final multivariable mixed model regressions of EQ-VAS for the full population (n = 285)

	Univariable regression		Multivariable regression	
	β (95% CI)	p-value	β (95% CI)	p-value
Constant	58.34 (50.81–65.89)	< 0.001	98.00 (74.04–121.70)	< 0.001
Intervention group	3.16 (−0.69–7.00)	0.108	2.06 (−1.38–5.50)	0.241
Time (reference: discharge)				
1 month	1.70 (−0.85–4.25)	0.191	1.35 (−1–20–3.90)	0.300
6 months	2.29 (−0.36–4.93)	0.090	1.84 (−0.80–4.48)	0.171
12 months	0.99 (−1.81–3.78)	0.489	0.41 (−2.39–3.20)	0.776
Study ward 1	**−10.35 (−14.83–−5.88)**	** < 0.001**	**−6.50 (−10.78–−2.22)**	**0.003**
Age when included^a^	**−0.67 (−0.98–−0.35)**	** < 0.001**	**−0.32 (−0.62–−0.01)**	**0.040**
Sex Female	−1.94 (−6.001–2.12)	0.348	NA	
Level of education > 12 years	1.00 (−3.07–5.07)	0.631	NA	
Home-dwelling before included	**7.04 (−1.54–15.62)**	**0.108**	NS	
Living alone before included	**−3.34 (−7.54–0.46)**	**0.083**	NS	
Home-care services	**−10.57 (−14.34–−6.81)**	** < 0.001**	**−3.99 (−7.87–−0.11)**	**0.044**
Number of medications total^a^	**−1.20 (−1.53–−0.86)**	** < 0.001**	**−1.00 (−1.32–−0.66)**	** < 0.001**
Handling own medications	**8.83 (4.96–12.70)**	** < 0.001**	NS	
Multidose adherence aid	**−10.75 (−14.98–−6.52)**	** < 0.001**	NS	
Hypertension	1.35 (−2.62–5.32)	0.506	NA	
Asthma or COPD	−**2.61 (**−**6.91–1.70)**	**0.235**	NS	
Atrial fibrillation	−**3.60 (**−**8.13–0.92)**	**0.119**	NS	
Diabetes	−**3.74 (**−**8.59–1.11)**	**0.131**	NS	
Heart failure	−**8.27 (**−**13.86–**−**2.67)**	**0.004**	NS	
Renal failure	−1.57 (−6.95–3.81)	0.567	NA	
Anxiety / depression	−**5.48 (**−**12.46–1.49)**	**0.123**	NS	
Dementia	−**1.98 (**−**14.58–10.62)**	**0.758**	NS	
Charlson Comorbidity Index^b^	−**1.17 (**−**2.19–**−**0.16)**	**0.023**	NS	

*COPD* Chronic Obstructive Pulmonary Disease; *CI* confidence interval; *β* regression coefficient; *NS* not significant in multivariate regression; *NA* not applicable based on univariate regression

^a^ Continuous varable

^b^ Tested in a separate model from other comorbidities

Results in bold are statistically significant

The number of medications and age were associated with decreasing EQ-VAS (−1.00 [−1.32–−0.66], *p* < *0.001* and −0.32 [−0.62–−0.01], *p* = *0.040*, respectively). Being admitted to study ward 1 and receiving home-care services were also associated with reduced EQ-VAS (−6.50 [−10.78–−2.22], *p* = *0.003* and −3.99 [−7.78–−0.11], *p* = *0.044*, respectively). None of the comorbidities, CCI nor sex were significant in the multivariable model.

## Discussion

### Main findings

The EQ-5D-3L index scores of older adults ≥ 70 years at discharge from an acute hospital admission was 0.518 and 0.472 for the intervention and control groups, respectively. For the full population and long-stayers, the EQ-5D-3L index scores deteriorated throughout the study period. For the non-long stayer subgroup, the EQ-5D-3L index scores significantly improved after 1 month and showed no significant decrease. The main factor associated with lower HRQoL was the number of medications. Being home-dwelling was the main factor associated with higher HRQoL, and receiving home-care services was negatively associated. Sex and comorbidities contributed to a lesser extent in the multivariate regression models. No significant differences between the two IMMENSE study groups were observed. However, the intervention group reported higher EQ-VAS compared to the control group, which was statistically significant in the non-long stayer subgroup. Although there are some deviations, findings are overall consistent between the dimensions, EQ-5D-3L index score, and EQ-VAS, with small differences between study groups and the same type of factors (i.e., medication burden and care needs) influencing outcomes.

The study population had a substantially impaired HRQoL throughout the study period compared to national and international population norms for adults ≥ 70 year [1, 27, 28]. The three dimensions with the highest prevalence of problems at discharge were Mobility (I: 75.0% and C: 76.6%), Usual Activities (I: 71.6% and C: 72.3%) and Pain/discomfort (I: 66.2% and C: 62.0%). This aligns with population norm studies, although with notably lower prevalence, ranging from 28–50% for Mobility, 17–37% for Usual activities, and 32–55% for Pain/Discomfort [1, 27, 28] for older adults ≥ 70 years. The EQ-5D-3L index scores at discharge (I:0.518 and C:0.472) were low compared to the Norwegian population norm of 0.786 [27]. Similarly, the EQ-VAS at discharge (I:59.16 and C:55.51) was low compared to population norms of 64.3–80.5 [1, 27, 28]. Even when comparing to older adults ≥ 85 years of age, the study population have impaired HRQoL [29]. In fact, our findings were more in line with reported results for pre-frail and frail persons after discharge from hospital following a stroke [30] or extended hospital care [31]. For the full population, the longitudinal change of HRQoL for the patients was a significant decrease in EQ-5D-3L index score from discharge to 12-months follow-up. The non-long stayers, however, experienced a significant increase in the EQ-5D-3L index scores after 1 month with no significant decrease throughout the follow-up. This may indicate that the non-long stayers were less frail than the long stayers.

The number of medications was the single factor with the strongest and most consistent association with reduced HRQoL, including problems across all five dimensions, reduced EQ-5D-3L index scores and reduced EQ-VAS. Polypharmacy was prevalent before and after the index hospital admission in our study population, with 81% and 89% using ≥ 5 medications at baseline and discharge, respectively. Even though the individual coefficients or ORs may appear small, they represent a reduction in HRQoL for each medication added to the treatment regimen. In fact, for a patient using the mean number of 9 medications, a twofold to sevenfold probability of problems in each of the five dimensions was present.^1^ Similarly, a reduction in the EQ-5D-3L index score of −0.18 and a reduction in the EQ-VAS of −9 points would apply.^2^ Moreover, in previous studies investigating the minimal clinically important difference (MID) of EQ-5D-3L, changes of 0.079–0.082 were viewed as clinically important [32, 33]. Notably, a trajectory analysis based on polypharmacy status, Aljeaidi et al. found that patients with incident polypharmacy had a steep decline in HRQoL, while patients with subsiding polypharmacy remained at a low HRQoL [5]. Our findings are also consistent with other studies reporting an association between polypharmacy and declining HRQoL [2–4], and may reflect that older adults exposed to polypharmacy are at greater risk of ADEs and ADRs compromising their HRQoL.

Being home-dwelling was associated with higher HRQoL, in terms of lower prevalence of reported problems in the dimensions Mobility, Self-care, and Usual Activities. A subsequent higher EQ-5D-3L index score greater than MID was also identified, although no effect was seen on the EQ-VAS. This aligns with the results from a German study in older adults ≥ 85 years [29]. We also observed that receiving home-care services had the opposite effect in the same three dimensions, EQ-5D-3L index scores, and EQ-VAS. Receiving multidose adherence aid, similarly, was associated with lower EQ-5D-3L-index, greater than MID. The combined findings contribute to the growing body of evidence highlighting the importance of physical functioning for maintaining a good HRQoL [6, 34]. This is also consistent with the findings of Montiel-Luque et al. indicating that patients reporting no problems in Mobility had the highest EQ-VAS scores, while those reporting problems with in Self-care the lowest EQ-VAS scores [3].

None of the comorbidities nor the CCI were significantly associated with EQ-5D-3L index scores or EQ-VAS in multivariable regressions, although some comorbidities were associated with some of the dimensions. This contrasts with findings from other studies where comorbidities or multimorbidity were significantly associated with reduced HRQoL [1, 6]. However, it is worth noting that these studies did not including information about the number of medications, living arrangements and home-care services in their models. In the multivariable model, the direction of effect of heart failure was inversed compared to the univariable model, indicating reduced problems with usual activities. This may indicate some dependency among the covariates, although not enough to cause variables being omitted.

Age and sex are often considered confounding factors in HRQoL studies. However, in the present study they were not consistently associated with HRQoL. Age was associated with three of the dimensions with an 8% increased odds per year of reporting problems in Mobility and Self-care, and a 7% reduced odds of reporting Anxiety/Depression. For the EQ-5D-3L index scores and EQ-VAS, age was associated with reductions of -0.003 and -0.32 per year, respectively. Our findings thus suggest that adjusting for age could be relevant. Female sex was only associated with two of the dimensions (Pain/Discomfort and Anxiety/Depression) in our study and was not significantly associated with EQ-5D-3L index scores nor EQ-VAS. Hence, other more strongly associated factors were more important for HRQoL in our population.

The only significantly impact of the intervention on HRQoL was observed in the non-long stayer group in relation to the EQ-VAS, reflecting a higher subjectively reported HRQoL. Romskaug et al. [4], investigating HRQoL among home-dwelling persons ≥ 70 years after a medication review intervention, reported a significantly improved HRQoL measured using the 15D instrument 16 weeks after the intervention. Their study population differed from ours as they were not recruited during an acute hospitalization and were likely less frail. The fact that approximately 60% of our study population reported moderate or extreme pain throughout the 12-month study period, suggests that they may not have received adequate pain management. The finding aligns with the Norwegian population norm [27]. It is plausible that greater attention to pain management could have contributed to an enhanced HRQoL in the intervention group.

### Strengths and limitations of the study

The major strength of this study is that it builds on an RCT with high completeness of HRQoL data and a longitudinal design with a 12-months follow-up. We have also been able to include covariates of age, sex, living arrangements, home-care services, medications, and comorbidities. Many studies on HRQoL in older adults have a cross-sectional design [1–3, 27–29, 34–37], offering only a snapshot of the situation. Others with longitudinal designs often include fewer measurement points and fewer covariates [4–6, 30, 31, 38].

However, the findings must be interpreted with some limitations in mind. First, due to the acute care setting, HRQoL measurements were not carried out at baseline in the study. As a results, all regression analyses are done with reference to discharge from the index hospitalization, after initiating the intervention. Although not statistically significant, the intervention group displayed a higher mean EQ-5D-3L index score and higher mean EQ-VAS at discharge compared to the control group. We cannot ascertain if this is an effect of the intervention, or if the intervention group had higher HRQoL before discharge, or even before hospitalization. If we had known the pre-randomization HRQoL of the included patients, we could have had more reliable results with respect to the effect of the intervention. Second, the analysis was conducted on a sub-set of the randomized population. Excluding the patients for whom next of kin provided informed consent reduces an already small sample size, limiting the possibility of finding statistically significant between-group differences and the generalizability of the results to all older adults. Thus, future studies should consider using proxy assessments to enable HRQoL reporting for all included patients. However, the distribution of intervention and control patients is equal between the present study population and the entire IMMENSE population [18]. This enables comparison between the two study groups. Third, the comorbidities reported in this study were based on the hospital admission and discharge notes and are therefore likely incomplete. More detailed records of comorbidities could have produced a more reliable results of associations between comorbidities or CCI, and HRQoL. Fourth, the heterogeneity of the study population was addressed in a subgroup analysis based on length of stay. To some extent, the categorization based on length of stay serves as a proxy for frailty in this study population. Performing formal frailty assessments could have provided more insight into the heterogeneity of the studied patient group, and hence been a more reliable basis for subgroup categorization than the length of stay. Fifth, the most important comparisons in our analysis were the between-group differences in EQ-5D-3L index scores and EQ-VAS for the full population. All other reported results of changes over time and associated factors were part of a preplanned model building strategy to find this difference. The subgroup analyses should be interpreted as exploratory analyses. Finally, the analysis method assumes missing at random even though factors causing non-response may be present. However, a previous study indicated that mixed model regression without previous multiple imputation could handle this level of missingness [23].

### Implications and need for future research

Although we did not observe longitudinal improvements in HRQoL in the current study, a previously published cost-utility analysis reported that the intervention produced more quality adjusted life years (QALYs) than standard care [18]. The reported factors associated with impaired HRQoL in the current study (increasing number of medications and the need for home-care services), are plausible, as deteriorating health could both affect the associated factors and HRQoL. Furthermore, reported comorbidities were not associated with HRQoL in the presence of other associated factors. The crucial question that remains as to how to enhance or prevent deterioration in health and HRQoL among older adults. A growing body of evidence suggests that medication management interventions can reduce medication-related hospital readmissions [12]. We identified a high prevalence of pain, which has the potential directly influence HRQoL, and should be addressed as an integral part of medication management. Further research is needed to explore how underlying factors, such as medication-related problems and potentially inappropriate medications are tied to drug-related morbidity, and how these factors influence the dimensions of HRQoL.

## Conclusion

We observed no significant difference in the longitudinal change in HRQoL between the two IMMENSE study groups over 12 months after hospitalization. However, the significantly increased 1-month EQ-5D-3L index scores combined with no significant decrease the remaining year in the non-long stayer subgroup indicates that the intervention may have had a long-term effect on HRQoL in some of intervention patients. The number of medications and the ability to live and care for oneself seems to be independent factors associated with HRQoL in the study population. This should be taken into consideration when planning future patient care and health-care services.

### Supplementary Information

Below is the link to the electronic supplementary material.

Supplementary file1 (PDF 1038 KB)

## Data Availability

The datasets generated and analyzed during the current study are not publicly available because they contain information that can compromise research participants’ privacy/consent but are available from the corresponding author on reasonable request.

## Footnotes

1 Anxiety/Depression dimension: OR1.09^9^ = 2.17 and Usual activities dimension: OR1.24.^9^ = 6.93.

2 EQ-5D-3L index score: β-0.02 × 9 = −0.18 and EQ-VAS: β-1.00 × 9 = −9.
