# Supplementary material for: Health-related quality of life among older adults following acute hospitalization: longitudinal analysis of a randomized controlled trial
Source: Qual Life Res. 2024 Jun 17;33(8):2219–33. doi: 10.1007/s11136-024-03689-x (PMC11286627; doi:10.1007/s11136-024-03689-x)
Supplement: Supplementary file 1 — Supplementary file1 (PDF 1038 KB) [file 11136_2024_3689_MOESM1_ESM.pdf]

Supplementary Information

Health-related quality of life among older adults following acute hospitalization

Journal

Quality of Life Research

Authors

Eirin Guldsten Robinson[1], Hanna Gyllensten[2], Anne Gerd Granas[1], Kjell H Halvorsen[3], Beate Hennie Garcia [3,4]

Affiliations

[1] Department of Pharmacy, University of Oslo, 0316 Oslo, Norway.

[2] Institute of Health and Care Sciences, Sahlgrenska Academy, University of Gothenburg, Gothenburg, Sweden

[3] Department of Pharmacy, Faculty of Health Sciences, UiT the Arctic University of Norway, Tromsø, Norway.

[4] Hospital Pharmacy of North Norway Trust, Tromsø, Norway

Correspondence to: e.g.robinson@farmasi.uio.no

Table S1 Problems in the EQ-5D dimensions, index scores and EQ-VAS in the non-long stayers (n=222) 2

Table S2 Problems in the EQ-5D dimensions, index scores and EQ-VAS in the long stayers (n=63) 3

Table S3 Results of multivariable multi-level logistic regression models of EQ-5D dimensions (no problem versus problem (moderate or extreme)) for the non-long stayers (n=222) 4

Table S4 Results of multivariable multi-level logistic regression models of EQ-5D dimensions (no problem versus problem (moderate or extreme)) for the long stayers (n=63) 5

Table S5 Results of univariable multi-level logistic regression models of EQ-5D dimensions (no problem versus problem (moderate or extreme)) for the full population (n=285) 6

Table S6 Results of univariable multi-level logistic regression models of EQ-5D dimensions (no problem versus problem (moderate or extreme)) for the non-long stayers (n=222) 7

Table S7 Results of univariable multi-level logistic regression models of EQ-5D dimensions (no problem versus problem (moderate or extreme)) for the long stayers (n=63) 8

Table S8 Results of univariable and final multivariable mixed model regressions of index scores for the non-long stayers (n=222) and long stayers (n=63) 9

Table S9 Results of univariable and final multivariable mixed model regressions of EQ-5D VAS for the non-long stayers (n=222) and long stayers (n=63) 10

Table S1 Problems in the EQ-5D dimensions, index scores and EQ-VAS in the non-long stayers (n=222)

	Discharge N=222		1 month N=217		6 months N=211		12 months N=205	
	**Intervention group n=104**	**Control group n=116**	**Intervention group n=105**	**Control group n=112**	**Intervention group n=101**	**Control group n=110**	**Intervention group n=99**	**Control group n=106**
**Mobility, n (%)**								
No problems	29 (27.4)	31 (26.7)	34 (32.4)	28 (25.0)	32 (31.7)	21 (19.1)	26 (26.3)	16 (15.1)
Moderate problems	68 (64.2)	75 (64.7)	62 (59.1)	70 (62.5)	60 (59.4)	70 (63.6)	58 (58.6)	67 (63.2)
Extreme problems	7 (6.6)	10 (8.6)	6 (5.7)	6 (5.4)	3 (3.0)	8 (7.3)	6 (6.1)	8 (7.6)
Reporting any problems[a]	75 (70.8)	85 (73.3)	68 (64.8)	76 (67.9)	63 (62.4)	78 (70.9)	64 (64.7)	75 (70.8)
Missing	2 (1.9)	0 (0.0)	3 (2.9)	8 (7.1)	6 (5.9)	11 (10.0)	9 (9.1)	15 (14.2)
**Self-care, n (%)**								
No problems	69 (65.1)	74 (63.8)	70 (66.7)	74 (66.1)	78 (77.2)	75 (68.2)	64 (64.7)	69 (65.1)
Moderate problems	28 (26.4)	30 (25.9)	28 (26.7)	24 (21.4)	15 (14.9)	17 (15.5)	24 (24.2)	14 (13.2)
Extreme problems	7 (6.6)	12 (10.3)	4 (3.8)	6 (5.4)	2 (2.0)	6 (5.5)	2 (2.0)	8 (7.6)
Reporting any problems[a]	35 (33.0)	42 (36.2)	32 (30.5)	30 (26.8)	17 (16.8)	23 (20.9)	26 (26.3)	22 (20.8)
Missing	2 (1.9)	0 (0.0)	3 (2.9)	8 (7.1)	6 (5.9)	12 (10.9)	9 (9.1)	15 (14.2)
**Usual activities, n (%)**								
No problems	32 (30.2)	35 (30.2)	42 (40.0)	29 (25.9)	34 (33.7)	29 (36.4)	34 (34.3)	28 (26.4)
Moderate problems	44 (41.5)	46 (39.7)	43 (41.0)	53 (47.3)	46 (45.5)	47 (42.7)	40 (40.4)	41 (38.7)
Extreme problems	28 (26.4)	35 (30.2)	17 (16.2)	22 (19.6)	15 (14.9)	21 (19.1)	16 (16.2)	22 (20.8)
Reporting any problems[a]	72 (67.9)	81 (69.8)	60 (57.1)	75 (67.0)	61 (60.4)	68 (61.8)	56 (56.6)	63 (59.4)
Missing	2 (1.9)	0 (0.0)	3 (2.9)	8 (7.1)	6 (5.9)	13 (11.8)	9 (9.1)	15 (14.2)
**Pain/discomfort, n (%)**								
No problems	33 (31.1)	42 (36.2)	34 (32.4)	44 (39.3)	36 (35.6)	30 (27.3)	26 (26.3)	27 (25.5)
Moderate problems	58 (54.7)	57 (49.1)	57 (54.3)	49 (43.8)	48 (47.5)	50 (45.5)	53 (53.5)	49 (46.2)
Extreme problems	13 (12.3)	17 (14.7)	11 (10.5)	11 (9.8)	11 (10.9)	18 (16.4)	11 (11.1)	15 (14.2)
Reporting any problems[a]	71 (67.0)	74 (63.8)	68 (64.8)	60 (53.6)	59 (58.4)	68 (61.8)	64 (64.6)	64 (60.4)
Missing	2 (1.9)	0 (0.0)	3 (2.9)	8 (7.1)	6 (5.9)	12 (10.9)	9 (9.1)	15 (14.2)
**Anxiety/depression, n (%)**								
No problems	63 (59.4)	68 (58.6)	63 (60.0)	67 (59.8)	59 (58.4)	56 (51.0)	55 (55.6)	57 (53.8)
Moderate problems	35 (33.0)	39 (33.6)	36 (34.3)	32 (28.6)	35 (34.7)	37 (33.6)	34 (34.3)	29 (27.4)
Extreme problems	6 (5.7)	9 (7.8)	3 (2.9)	4 (3.6)	1 (1.0)	5 (4.6)	1 (1.0)	4 (3.8)
Reporting any problems[a]	41 (38.7)	48 (41.4)	39 (37.1)	36 (32.1)	36 (35.6)	42 (38.2)	35 (35.4)	33 (31.1)
Missing	2 (1.9)	0 (0.0)	3 (2.9)	9 (8.0)	6 (5.9)	12 (10.9)	9 (9.1)	16 (15.1)
**Any dimension, n (%)**								
No problems	8 (7.6)	12 (10.3)	11 (10.5)	12 (10.7)	12 (11.9)	8 (7.3)	5 (5.1)	9 (8.5)
Moderate problems	57 (53.8)	55 (47.4)	64 (61.0)	62 (55.4)	57 (56.4)	58 (52.7)	59 (59.6)	52 (49.1)
Extreme problems	39 (36.8)	49 (42.2)	27 (25.7)	30 (26.8)	26 (25.7)	33 (30.0)	26 (26.3)	30 (28.3)
Reporting any problems[a]	96 (90.6)	104 (89.7)	91 (86.7)	92 (82.1)	83 (82.2)	91 (82.7)	85 (85.9)	82 (77.4)
Missing	2 (1.9)	0 (0.0)	3 (2.9)	8 (7.1)	6 (5.9)	11 (10.0)	9 (9.1)	15 (14.2)
**EQ-5D-3L index score[b]**	0.511	0.491	0.567	0.529	0.559	0.451	0.501	0.436
**EQ-5D-3L VAS score[b]**	60.29	56.27	61.86	60.71	64.08	58.92	62.87	57.41

[a]Reporting any problems is the sum of moderate problems and extreme problems.

[b]Unadjusted

Abbreviations: EQ-VAS, EuroQol visual analogue scale

Table S2 Problems in the EQ-5D dimensions, index scores and EQ-VAS in the long stayers (n=63)

	Discharge N=63		1 month N=60		6 months N=57		12 months N=54	
	**Intervention group n=42**	**Control group n=21**	**Intervention group n=40**	**Control group n=20**	**Intervention group n=38**	**Control group n=19**	**Intervention group n=35**	**Control group n=19**
**Mobility, n (%)**								
No problems	6 (14.3)	1 (4.8)	7 (17.5)	1 (5.0)	3 (7.9)	2 (10.5)	4 (11.1)	2 (10.5)
Moderate problems	33 (78.6)	17 (81.0)	24 (60.0)	16 (80.0)	23 (60.5)	15 (79.0)	14 (38.9)	12 (63.2)
Extreme problems	3 (7.1)	3 (14.3)	2 (5.0)	1 (5.0)	1 (2.6)	0 (0.0)	4 (11.1)	1 (5.3)
Reporting any problems[a]	36 (85.7)	20 (95.2)	26 (65.0)	17 (85.0)	24 (63.2)	15 (79.0)	18 (50.0)	13 (68.4)
Missing	0 (0.0)	0 (0.0)	7 (17.5)	2 (10.0)	11 (29.0)	2 (10.5)	14 (38.9)	4 (21.1)
**Self-care, n (%)**								
No problems	24 (57.1)	9 (42.9)	17 (42.5)	11 (55.0)	17 (44.7)	9 (47.4)	13 (36.1)	9 (47.4)
Moderate problems	15 (35.7)	6 (28.6)	14 (35.0)	5 (25.0)	7 (18.4)	7 (36.8)	5 (13.9)	5 (26.3)
Extreme problems	3 (7.1)	6 (28.6)	2 (5.0)	2 (10.0)	3 (7.9)	1 (5.3)	4 (11.1)	1 (5.3)
Reporting any problems[a]	18 (42.9)	12 (57.1)	16 (40.0)	7 (35.0)	10 (26.3)	8 (42.1)	9 (25.0)	6 (31.6)
Missing	0 (0.0)	0 (0.0)	7 (17.5)	2 (10.0)	11 (29.0)	2 (10.5)	14 (38.9)	4 (21.1)
**Usual activities, n (%)**								
No problems	8 (19.1)	3 (14.3)	6 (15.0)	1 (5.0)	4 (10.5)	2 (10.5)	4 (11.1)	1 (5.3)
Moderate problems	22 (52.4)	8 (38.1)	14 (35.0)	8 (40.0)	15 (39.5)	12 (63.2)	11 (30.6)	11 (57.9)
Extreme problems	12 (28.6)	10 (47.6)	13 (32.5)	9 (45.0)	8 (21.1)	3 (15.8)	7 (19.4)	3 (15.8)
Reporting any problems[a]	34 (81.0)	18 (85.7)	27 (67.5)	17 (85.0)	23 (60.5)	15 (79.0)	18 (50.0)	14 (73.7)
Missing	0 (0.0)	0 (0.0)	7 (17.5)	2 (10.0)	11 (29.0)	2 (10.5)	14 (38.9)	4 (21.1)
**Pain/discomfort, n (%)**								
No problems	15 (35.7)	10 (47.6)	9 (22,5)	4 (20.0)	9 (23.7)	4 (21.1)	6 (16.7)	2 (10.5)
Moderate problems	22 (52.4)	9 (42.9)	20 (50.0)	11 (55.0)	13 (34.2)	10 (52.6)	10 (27.8)	10 (52.6)
Extreme problems	5 (11.9)	2 (9.5)	4 (10.0)	3 (15.0)	5 (13.2)	2 (10.5)	6 (16.7)	3 (15.8)
Reporting any problems[a]	27 (64.3)	11 (52.4)	24 (60.0)	14 (70.0)	18 (47.4)	12 (63.2)	16 (44.4)	13 (68.4)
Missing	0 (0.0)	0 (0.0)	7 (17.5)	2 (10.0)	11 (29.0)	3 (15.8)	14 (38.9)	4 (21.1)
**Anxiety/depression, n (%)**								
No problems	33 (78.6)	8 (38.1)	23 (57.5)	11 (55.0)	16 (42.1)	8 (42.1)	15 (41.7)	8 (42.1)
Moderate problems	7 (16.7)	11 (52.4)	8 (20.0)	5 (25.0)	9 (23.7)	7 (36.8)	4 (11.1)	7 (36.8)
Extreme problems	2 (4.8)	2 (9.5)	2 (5.0)	2 (10.0)	2 (5.3)	1 (5.3)	3 (8.3)	0 (0.0)
Reporting any problems[a]	9 (21.4)	13 (61.9)	10 (25.0)	7 (35.0)	11 (29.0)	8 (42.1)	7 (19.4)	7 (36.8)
Missing	0 (0.0)	0 (0.0)	7 (17.5)	2 (10.0)	11 (29.0)	3 (15.8)	14 (38.9)	4 (21.1)
**Any dimension, n (%)**								
No problems	3 (7.1)	0 (0.0)	3 (7.5)	0 (0.0)	3 (7.9)	0 (0.0)	2 (5.6)	0 (0.0)
Moderate problems	22 (52.4)	9 (42.9)	14 (35.0)	6 (30.0)	12 (31.6)	12 (63.2)	9 (25.0)	9 (47.4)
Extreme problems	17 (40.5)	12 (57.1)	16 (40.0)	12 (60.0)	12 (31.6)	5 (26.3)	11 (30.6)	6 (31.6)
Reporting any problems[a]	39 (92.9)	21 (100.0)	30 (75.0)	18 (90.0)	24 (63.2)	17 (89.5)	20 (55.6)	15 (79.0)
Missing	0 (0.0)	0 (0.0)	7 (17.5)	2 (10.0)	11 (29.0)	2 (10.5)	14 (38.9)	4 (21.1)
**EQ-5D-3L index score[b]**	0.508	0.366	0.394	0.330	0.342	0.459	0.242	0.362
**EQ-5D-3L VAS score[b]**	56.15	51.83	52.85	49.12	52.72	58.53	52.97	54.85

[a]Reporting any problems is the sum of moderate problems and extreme problems.

[b]Unadjusted

Abbreviations: EQ-VAS, EuroQol visual analogue scale

Table S3 Results of multivariable multi-level logistic regression models of EQ-5D dimensions (no problem versus problem (moderate or extreme)) for the non-long stayers (n=222)

	Mobility		Self-care		Usual activities		Pain/Discomfort		Anxiety/Depression	
	**OR (95% CI)**	**p-value**	**OR (95% CI)**	**p-value**	**OR (95% CI)**	**p-value**	**OR (95% CI)**	**p-value**	**OR (95% CI)**	**p-value**
Constant	0.32 (0.10 – 1.02)	0.055	0.11 (0.02 – 0-79)	0.029	0.00 (0.00 – 0.01)	<0.001	0.55 (0.26 – 1.18)	0.127	0.08 (0.03 – 0.20)	<0.001
Intervention group	0.61 (0.26 – 1.42)	0.253	1.10 (0.53 – 2.27)	0.793	0.70 (0.03 – 1.47)	0.352	1.12 (0.62 – 2.00)	0.707	0.93 (0.43 – 2.04)	0.860
Time (reference: discharge)										
1 month	0.81 (0.44 – 1.49)	0.499	0.72 (0.41 – 1.29)	0.271	0.75 (0.42 – 1.34)	0.331	0.78 (0.47 – 1.28)	0.328	0.77 (0.44 – 1.36)	0.369
6 months	1.18 (0.62 – 2.21)	0.616	**0.31 (0.16 – 0.59)**	**<0.001**	0.95 (0.52 – 1.73)	0.858	0.98 (0.59 – 1.62)	0.924	1.17 (0.66 – 2.07)	0.593
12 months	2.14 (1.09 – 4.17)	0.026	0.72 (0.39 – 1.32)	0.286	0.99 (0.54 – 1.81)	0.965	1.40 (0.82 – 2.39)	0.219	0.98 (0.55 – 1.77)	0.957
Study ward 1	**4.08 (1.54 – 10.76)**	**0.005**	**2.49 (0.00 – 6.22)**	**0.050**	**2.57 (1.07 – 6.16)**	**0.035**	NS		NS	
Age when included[a]	NS		NS		**1.10 (1.03 – 1.18)**	**0.004**	NS		NA	
Sex Female	NS		NA		NS		**2.54 (1.39 – 4.65)**	**0.002**	**3.19 (1.41 – 7.21)**	**0.005**
Level of education >12 years	NA		**2.26 (1.08 – 4.71)**	**0.030**	NA		NA		NA	
Home-dwelling before included	NS		**0.10 (0.02 – 0.53)**	**0.006**	NS		NA		NS	
Living alone before included	NS		NS		NS		NA		NA	
Home-care services	**6.17 (2.37 – 16.09)**	**<0.001**	**12.59 (5.26 – 30.14)**	**<0.001**	**3.49 (1.52 – 8.04)**	**0.003**	NS		NS	
Number of medications total[a]	**1.17 (1.07 – 1.29)**	**0.001**	**1.09 (1.01 – 1.17)**	**0.018**	**1.26 (1.15 – 1.37)**	**<0.001**	**1.12 (1.06 – 1.19)**	**<0.001**	NS	
Handling own medications	NS		NS		NS		NA		NS	
Multidose adherence aid	NS		NS		NS		NA		**2.89 (1.18 – 7.06)**	**0.020**
Hypertension	NA		NA		NA		NS		NA	
Asthma or COPD	NA		NS		NS		NS		NA	
Atrial fibrillation	NS		NS		NS		NA		**2.50 (1.03 – 6.05)**	**0.042**
Diabetes	NS		NS		NS		NA		**2.70 (1.00 – 7.25)**	**0.049**
Heart failure	NS		NS		NS		NA		NS	
Renal failure	NA		NA		NA		NA		NS	
Anxiety / depression	NA		NA		NA		NS		**28.27 (6.36 – 125.53)**	**<0.001**
Dementia	NA		NS		NA		**0.08 (0.01 – 0.49)**	**0.007**	NA	
Charlson Comorbidity Index[b]	NS		NS		NS		NA		NA	

[a] Continuous varable

[b] Tested in a separate model from other comorbidities.

Abbreviations: COPD, Chronic Obstructive Pulmonary Disease; OR, odds ratio; CI, confidence interval; NS, not significant in multivariate regression; NA, not applicable based on univariate regression.

Table S4 Results of multivariable multi-level logistic regression models of EQ-5D dimensions (no problem versus problem (moderate or extreme)) for the long stayers (n=63)

	**Mobility**		**Self-care**		**Usual activities**		**Pain/Discomfort**		**Anxiety/Depression**	
	**OR (95% CI)**	**p-value**	**OR (95% CI)**	**p-value**	**OR (95% CI)**	**p-value**	**OR (95% CI)**	**p-value**	**OR (95% CI)**	**p-value**
Constant	7.25 (0.74 – 70.56)	0.088	0.05 (0.01 – 0.35)	0.002	0.84 (0.09 – 8.26)	0.882	1.31 (0.37 – 4.67)	0.677	0.04 (0.00 – 0.35)	0.004
Intervention group	0.57 (0.11 – 2.99)	0.502	1.13 (0.33 – 3.89)	0.842	0.54 (0.10 – 2.97)	0.482	1.09 (0.46 – 2.61)	0.843	0.25 (0.05 – 1.19)	0.082
Time (reference: discharge)										
1 month	0.55 (0.14 – 2.11)	0.382	0.95 (0.35 – 2.56)	0.914	1.54 (0.38 – 6.20)	0.544	2.23 (0.90 – 5.56)	0.085	0.89 (0.28 – 2.82)	0.843
6 months	0.97 (0.22 – 4.25)	0.963	0.70 (0.24 – 2.02)	0.511	1.57 (0.36 – 6.76)	0.548	1.82 (0.71 – 4.66)	0.213	2.39 (0.72 – 7.94)	0.155
12 months	0.62 (0.14 – 2.67)	0.519	1.00 (0.32 – 3.08)	0.995	1.94 (0.40 – 9.41)	0.410	3.37 (1.13 – 10.00)	0.029	1.37 (0.39 – 4.82)	0.623
Study ward Ward 1	NA		NS		**21.28 (2.30 – 196.75)**	**0.007**	NA		NA	
Age when included[a]	NS		NS		NS		NS		NA	
Sex Female	NA		NA		NA		NA		NS	
Level of education >12 years	NA		NS		NS		NA		NA	
Home-dwelling before included	NS		NA		NA		NA		NA	
Living alone before included	NA		NA		NS		NA		NA	
Home-care services	NS		**6.5 (1.68 – 25.22)**	**0.007**	NS		NA		NS	
Number of medications total[a]	**1.20 (1.01 – 1.42)**	**0.040**	**1.17 (1.03 – 1.34)**	**0.014**	NS		**1.11 (1.02 – 1.22)**	**0.015**	**1.32 (1.12 – 1.57)**	**0.001**
Handling own medications	NA		NS		NS		NA		NS	
Multidose adherence aid	NA		NS		NS		NA		NS	
Hypertension	NA		NA		NS		**0.40 (0.17 – 0.91)**	**0.029**	NS	
Asthma or COPD	NA		NA		**40.29 (1.42 – 1144.25)**	**0.030**	NS		NS	
Atrial fibrillation	NA		NS		NA		**0.31 (0.12 – 0.78)**	**0.013**	NS	
Diabetes	NA		NA		NA		NA		NA	
Heart failure	NA		NS		NA		NA		NA	
Renal failure	NA		NS		NA		NA		NA	
Anxiety / depression	NA		NA		NA		NS		**16.71 (1.40 – 199.84)**	**0.026**
Dementia	NA		NA		NA		NA		NA	
Charlson Comorbidity Index[b]	NS		NS		NA		NA		NA	

[a] Continuous varable

[b] Tested separately from other comorbidities.

Abbreviations: COPD, Chronic Obstructive Pulmonary Disease; OR, odds ratio; CI, confidence interval; NS, not significant in multivariate regression; NA, not applicable based on univariate regression.

Table S5 Results of univariable multi-level logistic regression models of EQ-5D dimensions (no problem versus problem (moderate or extreme)) for the full population (n=285)

	**Mobility**		**Self-care**		**Usual activities**		**Pain/Discomfort**		**Anxiety/Depression**	
	**OR (95% CI))**	**p-value**	**OR (95% CI))**	**p-value**	**OR (95% CI))**	**p-value**	**OR (95% CI))**	**p-value**	**OR (95% CI))**	**p-value**
Constant	16.41 (7.31 – 36.84)	<0.001	0.36 (0.20 – 0.67)	0.001	10.02 (4.71 – 1.27)	<0.001	2.31 (1.46 – 3.65)	<0.001	0.48 (0.26 – 0.90)	0.022
Intervention group	0.60 (0.27 – 1.35	0.217	1.07 (0.52 – 2.20)	0.858	0.73 0.33 – 1.62)	0.435	1.16 (0.69 – 1.97)	0.588	0.64 (0.30 – 1.37)	0.250
Time (reference: discharge)										
1 month	0.69 (0.40 – 1.21)	0.199	0.73 (0.44 – 1.20)	0.215	0.74 (0.43 – 1.27)	0.274	0.99 (0.64 – 1.53)	0.968	0.80 (0.48 – 1.33)	0.392
6 months	1.02 (0.57 – 1.83)	0.948	0.36 (0.21 – 0.62)	<0.001	0.90 (0.51 – 1.58)	0.705	1.12 (0.72 – 1.76)	0.645	1.33 (0.79 – 2.22)	0.283
12 months	1.57 (0.85 – 2.90)	0.148	0.71 (0.42 – 1.21)	0.213	0.94 (0.53 – 1.67)	0.839	**1.68 (1.04 – 2.69)**	**0.034**	1.05 (0.62 – 1.78)	0.866
Study ward Ward 1	**9.08 (3.52 – 23.43)**	**<0.001**	**7.51 (3.00 – 18.81)**	**<0.001**	**11.33 (4.36 – 29.46)**	**<0.001**	0.91 (0.49 – 1.70)	0.772	**3.11 (1.27 – 7.61)**	**0.013**
Age when included (continuous)	**1.17 (1.09 – 1.25)**	**<0.001**	**1.10 (1.03 – 1.16)**	**0.003**	**1.20 (81.12 – 1.29)**	**<0.001**	0.99 (0.95 – 1.03)	0.752	**0.96 (0.90 – 1.02)**	**0.188**
Sex Female	1.47 (0.64 – 3.37)	0.367	0.91 (0.43 – 1.91)	0.806	**2.03 (0.89 – 4.66)**	**0.093**	**2.34 (1.36 – 4.03)**	**0.002**	**3.67 (1.68 – 8.03)**	**0.001**
Level of education >12 years	1.03 (0.45 – 2.35)	0.939	1.08 (0.52 – 2.25)	0.826	**0.53 (0.23 – 1.21)**	**0.132**	1.09 (0.63 – 1.86)	0.765	0.70 (0.33 – 1.52)	0.369
Home-dwelling before included	**0.05 (0.00 – 0.60)**	**0.018**	**0.20 (0.05 – 0.89)**	**0.034**	**0.02 (0.00 – 0.37)**	**0.008**	0.69 (0.23 – 2.13)	0.523	**2.50 (0.54 – 11.63)**	**0.242**
Living alone before included	**2.77 (1.21 – 6.37**	**0. 016**	**2.30 (1.10 – 4.84)**	**0.027**	**4.83 (2.13 – 10.98)**	**<0.001**	1.20 (0.79 – 0.69)	0.519	1.16 (0.54 – 2.49)	0.703
Home-care services	**13.10 (5.59 – 30.67)**	**<0.001**	**17.44 (8.34 – 36.47)**	**<0.001**	**16.62 (7.17 – 38.52)**	**<0.001**	**1.62 (0.96 – 2.74)**	**0.071**	**3.33 (1.54 – 7.17)**	**0.002**
Number of medications total	**1.26 (1.15 – 1.38**	**<0.001**	**1.21 (1.13 – 1.30)**	**<0.001**	**1.37 (1.25 – 1.51)**	**<0.001**	**1.13 (1.08 – 1.19)**	**<0.001**	**1.17 (1.09 – 1.26)**	**<0.001**
Handling own medications	**0.10 (0.043 – 0.24)**	**<0.001**	**0.08 (0.04 – 0.18)**	**<0.001**	**0.06 (0.03 – 0.14)**	**<0.001**	1.05 (0.62 – 1.79)	0.854	**0.21 (0.10 – 0.46)**	**<0.001**
Multidose adherence aid	**6.62 ()2.53 – 17.32**	**<0.001**	**5.68 (2.57 – 12.54)**	**<0.001**	**12.19 (4.63 – 32.07)**	**<0.001**	**1.46 (0.82 – 2.61**	**0.201**	**4.89 (2.12 – 11.31)**	**<0.001**
Hypertension	0.96 (0.43 – 2.15)	0.916	0.98 (0.48 – 2.01)	0.959	1.42 (0.64 – 3.18)	0.390	1.20 (0.71 – 2.05)	0.489	0.67 (0.31 – 1.41)	0.289
Asthma or COPD	1.42 (0.58 – 3.49)	0.439	**1.72 (0.78 – 3.81)**	**0.178**	**3.32 (1.33 – 8.25)**	**0.010**	**1.92 (1.06 – 3.46)**	**0.030**	1.20 (0.52 – 2.76)	0.670
Atrial fibrillation	**2.83 (1.01 – 7.23**	**0.030**	**2.20 (0.98 – 4.91)**	**0.055**	**3.32 (1.32 – 8.32)**	**0.011**	1.08 (0.59 – 1.95)	0.807	**3.24 (1.38 – 7.58)**	**0.007**
Diabetes	**5.54 (1.82 -16.89)**	**0. 003**	**3.17 (1.30 – 7.78)**	**0.011**	**5.76 (1.94 – 17.06)**	**0.002**	1.09 (0.56 -2.10)	0.808	**3.01 (1.14 – 7.93)**	**0.026**
Heart failure	**2.89 (0.88 – 9.47)**	**0. 079**	**4.01 (1.47 – 10.94)**	**0.007**	**2.50 (0.79 – 7.90)**	**0.117**	0.91 (0.43 – 1.91)	0.806	**2.03 (0.76 – 6.37)**	**0.144**
Renal failure	1.91 (0.61 – 6.07)	0.268	1.46 (0.54 – 3.93)	0.455	1.26 (0.41 – 3.84)	0.682	1.25 (0.60 – 2.61)	0.547	0.62 (0.22 – 1.76)	0.367
Anxiety / depression	1.20 (0.30 – 4.78)	0.793	**2.38 (0.70 – 8.15)**	**0.167**	**2.40 (0.58 – 9.83)**	**0.225**	**2.60 (1.00 – 6.71)**	**0.048**	**43.18 (10.90 – 171.00)**	**<0.001**
Dementia	1.85 (0.19 – 18.47)	0.601	**7.83 (1.09 – 56.22)**	**0.041**	2.31 (0.23 – 22.68)	0.473	**0.17 (0.04 – 0.72)**	**0.016**	0.36 (0.05 – 2.89)	0.339
Charlson Comorbidity Index*	**1.50 (1.18 – 1.91)**	**<0.001**	**1.46 (1.20 – 1.77)**	**<0.001**	**1.41 (1.12 – 1.79)**	**0.004**	1.05 (0.92 – 1.21)	0.470	1.05 (0.86 – 1.29)	0.610

[a] Continuous varable

[b] Tested separately from other comorbidities.

Abbreviations: COPD, Chronic Obstructive Pulmonary Disease; OR, odds ratio; CI, confidence interval; NS, not significant in multivariate regression; NA, not applicable based on univariate regression.

Table S6 Results of univariable multi-level logistic regression models of EQ-5D dimensions (no problem versus problem (moderate or extreme)) for the non-long stayers (n=222)

	**Mobility**		**Self-care**		**Usual activities**		**Pain/Discomfort**		**Anxiety/Depression**	
	**Univariable regression OR (95% CI))**	**p-value**	**Univariable regression OR (95% CI))**	**p-value**	**Univariable regression OR (95% CI))**	**p-value**	**Univariable regression OR (95% CI))**	**p-value**	**Univariable regression OR (95% CI))**	**p-value**
Constant	12.59 (5.28 – 30.02)	<0.001	0.29 (0.15 – 0.58)	0.001	7.49 (3.44 – 16.29)	<0.001	2.58 (1.51 – 4.40)	<0.001	0.44 (0.22 – 0.87)	0.019
Intervention group	0.50 (0.20 – 1.26)	0.142	1.01 (0.44 – 2.31)	0.99	0.60 (0.25 – 1.46)	0.262	1.21 (0.64 – 2.29)	0.548	0.92 (0.40 – 2.14)	0.851
Time (reference: discharge)										
1 month	0.76 (0.41 – 1.40)	0.374	0.69 (0.39 – 1.23)	0.211	0.69 (0.38 – 1.24)	0.213	0.78 (0.47 – 1.28)	0.323	0.76 (0.43 – 1.34)	0.349
6 months	1.09 (0.58 – 2.05)	0.794	0.30 (0.16 – 0.57)	<0.001	0.86 (0.47 – 1.58)	0.629	0.97 (0.58 – 1.62)	0.911	1.14 (0.65 – 2.03)	0.636
12 months	1.96 (1.00 – 3.85)	0.049	0.67 (0.37 – 1.22)	0.192	0.89 (0.48 – 1.65)	0.715	1.39 (0.81 – 2.36)	0.232	0.98 (0.54 -1.75)	0.935
Study ward Ward 1	**9.61 (3.35 – 27.60)**	**<0.001**	**6.15 (2.24 – 16.91)**	**<0.001**	**8.57 (3.13 – 23.47)**	**<0.001**	1.01 (0.49 – 2.29)	0.989	**3.35 (1.27 – 8,82)**	**0.014**
Age when included (continuous)	**1.17 (1.08 – 1.27)**	**<0.001**	**1.07 (1.00 – 1.15)**	**0.047**	**1.21 (1.12 – 1.30)**	**<0.001**	1.01 (0.95 – 1.06)	0.813	0.97 (0.90 – 1.04)	0.370
Sex Female	**1.97 (0.76 – 5.06)**	**0.161**	1.00 (0.42 – 2.35)	0.994	**2.04 (0.82 – 5.05)**	**0.124**	**2.60 (1.56 – 5.63)**	**0.001**	**4.18 (1.74 – 10.05)**	**0.001**
Level of education >12 years	1.03 (0.41 – 2.63)	0.943	**1.66 (0.72 – 3.82)**	**0.237**	0.65 (0.26 – 1.60)	0.346	0.94 (0.49 – 1.78)	0.848	0.66 (0.28 – 1.56)	0.342
Home-dwelling before included	**0.05 (0.00 – 1.07)**	**0.056**	**0.12 (0.02 – 0.88)**	**0.038**	**0.05 (0.00 – 0.74)**	**0.030**	0.55 (0.11 – 2.69)	0.458	**0.29 (0.04 – 2.12)**	**0.220**
Living alone before included	**4.00 (1.54 – 10.40)**	**0.004**	**2.26 (0.96 – 5,32)**	**0.061**	**4.96 (2.02 – 12.21)**	**<0.001**	1.32 (0.69 – 2.52)	0.398	0.82 (0.35 – 1.91)	0.645
Home-care services	**16.69 (6.25 – 44.57)**	**<0.001**	**17.87 (7.65 – 41.72)**	**<0.001**	**16.12 (6.48 – 40.15)**	**<0.001**	**1.78 (0.95 – 3.34)**	**0.072**	**3.20 (1.36 – 7.55)**	**0.008**
Number of medications total	**1.28 (1.15 – 1.40)**	**<0.001**	**1.20 (1.11 – 1.31)**	**<0.001**	**1.37 (1.24 – 1.52)**	**<0.001**	**1.15 (1.08 – 1.22)**	**<0.001**	**1.12 (1.03 – 1.22)**	**0.006**
Handling own medications	**0.09 (0.03 – 0.23)**	**<0.001**	**0.08 (0.03 – 0.20)**	**<0.001**	**0.06 (0.02 – 0.15)**	**<0.001**	1.01 (0.53 – 1.90)	0.984	**0.26 (0.11 – 0.62)**	**0.002**
Multidose adherence aid	**9.49 (3.06 – 29.39)**	**<0.001**	**6.54 (2.57 – 16.67)**	**<0.001**	**13.40 (4.54 – 39.51)**	**<0.001**	1.45 (0.72 – 2.95)	0.301	**5.774 (2.19 – 15.06)**	**<0.001**
Hypertension	0.91 (0.36 – 2.25)	0.830	0.88 (0.38 – 2.02)	0.762	1.16 (0.48 – 2.80)	0.739	**1.63 (0.87 – 3.05)**	**0.129**	0.89 (0.38 – 2.06)	0.781
Asthma or COPD	1.50 (0.55 – 4.06)	0.429	**2.17 (0.88 – 5.34)**	**0.093**	**2.91 (1.10 – 7.75)**	**0.032**	**1.76 (0.88 – 3.52)**	**0.108**	0.77 (0.31 – 1.93)	0.578
Atrial fibrillation	**3.91 (1.34 – 11.41)**	**0.013**	**1.77 (0.70 – 4.47)**	**0.228**	**3.92 (1.43 – 10.77)**	**<0.001**	1.52 (0.74 – 3.09)	0.253	**3.33 (1.29 – 8.61)**	**0.013**
Diabetes	**6.81 (1.92 – 24.10)**	**0.003**	**3.18 (1.13 – 8.93)**	**0.029**	**4.17 (1.31 – 13.25)**	**0.016**	0.97 (0.44 – 2.14)	0-949	**3.50 (1.18 – 10.40)**	**0.024**
Heart failure	**3.25 (0.80 – 13.16)**	**0.098**	**3.75 (1.12 – 12.57)**	**0.032**	**2.65 (0.72 – 9.84)**	**0.145**	0.90 (0.36 – 2.26)	0.821	**1.82 (0.53 – 6.29)**	**0.343**
Renal failure	2.17 (0.55 – 8.61)	0.269	0 .82 (0.24 – 2.78)	0.746	0.97 (0.27 – 3.50)	0-959	1.01 (0.40 – 2.53)	0.980	**0.41 (0.12 – 1.41)**	**0.157**
Anxiety / depression	1.20 (0.25 – 5.79)	0.817	2.28 (0.54 – 9.56)	0.260	1.55 (0.33 – 7.25)	0.580	**2.34 (0.76 – 7.18)**	**0.137**	**45.38 (9.48 – 217,28)**	**<0.001**
Dementia	0.75 (0.05 – 11.36)	0.834	**9.43 (0.80 – 111.63)**	**0.075**	0.86 (0.06 – 11.88)	0.913	**0.07 (0.01 – 0.47)**	**0.006**	0.10 (0.01 – 1.53)	0.099
Charlson Comorbidity Index*	**1.48 (1.13 – 1.94)**	**0.004**	**1.42 (1.15 – 1.77)**	**0.001**	**1.40 (1.09 – 1.80)**	**0.008**	1.05 (0.89 – 1.23)	0.568	1.05 (0.85 – 1.31)	0.642

[a] Continuous varable

[b] Tested separately from other comorbidities.

Abbreviations: COPD, Chronic Obstructive Pulmonary Disease; OR, odds ratio; CI, confidence interval; NS, not significant in multivariate regression; NA, not applicable based on univariate regression.

Table S7 Results of univariable multi-level logistic regression models of EQ-5D dimensions (no problem versus problem (moderate or extreme)) for the long stayers (n=63)

	**Mobility**		**Self-care**		**Usual activities**		**Pain/Discomfort**		**Anxiety/Depression**	
	**Simple regression OR (95% CI))**	**p-value**	**Simple regression OR (95% CI))**	**p-value**	**Simple regression OR (95% CI))**	**p-value**	**Simple regression OR (95% CI))**	**p-value**	**Simple regression OR (95% CI))**	**p-value**
Constant	52.50 (6.43 – 428.30)	<0.001	0.99 (0.28 – 3.50)	0.992	40.04 (3.57 – 448.56)	0.003	1.79 (0.71 – 4.52)	0.217	0.91 (0.19 – 4.35)	0.906
Intervention group	0.39 (0.07 – 2.28)	0.295	0.76 (0.19 – 3.12)	0.704	0.50 (0.07 – 3.87)	0.508	0.93 (0.35 – 2.51)	0.892	0.15 (0.02 – 1.06)	0.057
Time (reference: discharge)										
1 month	0.53 (0.14 – 2.07)	0.360	0.89 (0.33 – 2.41)	0.817	1.31 (0.32 – 5.56)	0.708	2.25 (0.89 – 5.69)	0.086	0.93 (0.29 – 2.95)	0.897
6 months	0.92 (0.20 – 4.12)	0.911	0.66 (0.23 – 1.90)	0.438	1.38 (0.30 – 6.30)	0.681	1.75 (0.67 – 4.55)	0.250	2.35 (0.70 – 7.84)	0.166
12 months	0.58 (0.13 – 2.57)	0.475	0.94 (0.31 – 2.90)	0.917	1.71 (0.33 – 8.76)	0.521	3.23 (1.08 – 9.69)	0.036	1.31 (0.37 – 4.62)	0.670
Study ward Ward 1	2.78 (0.36 – 23.47)	0.326	**9.83 (1.07 – 89.84)**	**0.043**	**17.76 (1.72 – 183.44)**	**0.016**	0.44 (0.10 – 1.90)	0.273	2.33 (0.20 – 27.54)	0.503
Age when included (continuous)	**1.11 (0.99 – 1.26)**	**0.077**	**1.13 (1.01 – 1.26)**	**0.032**	**1.10 (0.94 – 1.28)**	**0.247**	**0.95 (0.87 – 1.02)**	**0.167**	0.92 (0.80 – 1.07)	0.279
Sex Female	0.47 (0.08 – 2.69)	0.393	0.84 (0.21 – 3.45)	0.810	2.73 (0.39 – 19.11)	0.311	1.08 (0.40 – 2.92)	0.882	**3.01 (0.51 – 17.61)**	**0.222**
Level of education >12 years	0.88 (0.18 – 4.29)	0.974	**0.27 (0.06 – 1.14)**	**0.074**	**0.16 (0.02 – 1.12)**	**0.065**	1.62 (0.62 – 4.28)	0.327	0.68 (0.11 – 4.03)	0.668
Home-dwelling before included	**0.15 (0.01 – 3.20)**	**0.222**	0.80 (0.11 – 5.78)	0.822	1 (omitted)		1.05 (0.26 – 4.28)	0.941	0.63 (0.05 – 7.32)	0.710
Living alone before included	0.42 (0.70 – 2.51)	0.340	2.00 (0.49 – 8.18)	0.332	**3.02 (0.46 – 19.74)**	**0.247**	0.80 (0.30 – 2.14)	0.658	1.25 (0.22 – 7.27)	0.803
Home-care services	**2.57 (0.54 – 12.30)**	**0.236**	**11.22 (2.68 – 46.93)**	**0.001**	**8.62 (1.29 – 57.47)**	**0.026**	1.22 (0.46 – 3.21)	0.693	**5.65 (0.88 – 36.09)**	**0.067**
Number of medications total	**1.20 (1.01 – 1.42)**	**0.040**	**1.24 (1.08 – 1.42)**	**0.002**	**1.33 (1.09 – 1.61)**	**0.005**	**1.09 (1.00 – 1.20)**	**0.062**	**1.37 (1.15 – 1.64)**	**0.001**
Handling own medications	0.41 (0.08 – 2.08)	0.283	**0.13 (0.03 – 0.50)**	**0.003**	**0.16 (0.02 – 1.13)**	**0.066**	1.33 (0.49 – 3.58)	0.576	**0.08 (0.01 – 0.054)**	**0.009**
Multidose adherence aid	1.02 (0.20 – 5.27)	0.984	**2.55 (0.63 – 10.30)**	**0.188**	**4.02 (0.52 – 30.71)**	**0.180**	1.46 (0.55 – 3.92)	0.448	**3.38 (0.60 – 19.20)**	**0.169**
Hypertension	1.10 (0.22 – 5.44)	0.907	1.29 (0.34 – 4.94)	0.710	**3.53 (0.50 – 25.11)**	**0.207**	**0.40 (0.16 – 0.99)**	**0.048**	**0.21 (0.04 – 1-21)**	**0.081**
Asthma or COPD	2.06 (0.31 – 13.76)	0.455	1.04 (0.22 – 5.06)	0.958	**32 15 (0.91 – 1138.49)**	**0.057**	**3.33 (1.07 – 10.40)**	**0.038**	**7.03 (0.98 – 50.45)**	**0.052**
Atrial fibrillation	0.70 (0.12 – 3.91)	0.682	**3.51 (0.77 – 15.97)**	**0.104**	1.22 (0.15 – 10.12)	0.850	**0.30 (0.14 – 1.08)**	**0.069**	**3.35 (0.47 – 23.66)**	**0.226**
Diabetes	2.26 (0.28 – 18 49)	0.448	2.53 (0.48 – 13.45)	0.275	1 (Empty)		1.44 (0.44 – 4.72)	0.547	1.36 (0.17 – 11.01)	0.773
Heart failure	1.17 (0.16 – 8.42)	0.879	**3.20 (0.60 – 17.00)**	**0.172**	0.98 (0.10 – 9.66)	0.984	0.93 (0.29 – 2.97)	0.898	5.22 (0.60 – 45.44)	0.134
Renal failure	0.67 (0.11 – 4.10)	0.666	**2.81 (0.58 – 13.7)**	**0.199**	1.16 (0.12 – 11.01)	0.895	1.91 (0.60 – 6.12)	0.273	1.90 (0.25 – 14.17)	0.533
Anxiety / depression	1.01 (0.08 – 12.77)	0.992	2.60 (0.27 – 24.04)	0.408	1 (Empty)		**3.77 (0.64 – 22.18)**	**0.142**	**45.18 (2.52 – 808.17)**	**0.010**
Dementia	1 (Empty)		4.63 (0.22 – 96.35)	0.322	1 (Empty)		0.96 (0.11 – 8.20)	0.971	6.06 (0.13 – 287.44)	0.360
Charlson Comorbidity Index*	**1.40 (0.86 – 2.30)**	**0.180**	**1.52 (0.97 – 2.36)**	**0.065**	1.27 (0.66 – 2.43)	0.470	1.09 (0.81 – 1.47)	0.576	0.99 (0.59 – 1.69)	0.984

[a] Continuous varable

[b] Tested separately from other comorbidities.

Abbreviations: COPD, Chronic Obstructive Pulmonary Disease; OR, odds ratio; CI, confidence interval; NS, not significant in multivariate regression; NA, not applicable based on univariate regression.

Table S8 Results of univariable and final multivariable mixed model regressions of index scores for the non-long stayers (n=222) and long stayers (n=63)

	**Non-long stayers (n=222)**				**Long stayers (n=222)**			
	**Univariable regression**		**Multivariable regression**		**Univariable regression**		**Multivariable regression**	
	**β (95% CI)**	**p-value**	**β (95% CI)**	**p-value**	**β (95% CI)**	**p-value**	**β (95% CI)**	**p-value**
Constant	0.47 (0.41 – 0.53)	<0.001	0.54 (0.37 – 0.70)	<0.001	0.46 (0.34 – 0.58)	<0.001	0.76 (0.61 – 0.90)	<0.001
Intervention group	0.06 (-0.02 – 0.13)	0.156	0.03 (-0.3 – 0.9)	0.363	0.003 (-0.12 – 0.13)	0.966	-0.05 (-0.15 – 0.05)	0.310
Time (reference: discharge)								
1 month	0.05 (0.003 – 0.09)	0.037	**0.05 (0.00 – 0.09)**	**0.040**	-0.09 (-0.19 – 0.1)	0.086	-0.08 (-0.18 – 0.02)	0.310
6 months	0.002 (-0.04 – 0.05)	0.925	0.001 (-0.04 – 0.05)	0.954	-0.08 (-0.118 – 0.02)	0.138	-0.08 (-0.18 – 0.03)	0.140
12 months	-0.03 (-0.09 – -0.01)	0.146	-0.03 (-0.08 – 0.01)	0.124	-0.17 (-.028 – -0.07)	0.001	**-0.17 (-0.27 – -0.07)**	**0.001**
Study ward 1	**-0.16 (-0.25 – 0.13)**	**0.117**	NS		**-0.11 (-0.29 – 0.06)**	**0.206**	NS	
Age when included[a]	**-0.01 (-0.02 – -0.01)**	**0.001**	NS		-0.004 (-0.02 – 0.01)	0.362	NA	
Sex Female	**-0.05 (-0.13 – 0.03)**	**0.225**	NS		**-0.08 (-0.21 – 0.04)**	**0.192**	NS	
Level of education >12 years	-0.01 (-0.09 – 0.07)	0.800	NA		**0.12 (0.00 – 0.24)**	**0.049**	NS	
Home-dwelling before included	**0.27 (-0.09 – 0.44)**	**0.003**	**0.23 (0.08 – 0.38)**	**0.003**	0.05 (-0.13 – 0.23)	0.593	NA	
Living alone before included	-0.03 (-0.11 – 0.05)	0.417	NA		0.01 (-0.11 – 0.14)	0.903	NA	
Home-care services	**-0.26 (-0.33 – -0.19)**	**<0.001**	**-0.15 (-0.23 – -0.07)**	**<0.001**	**-0.20 (-0.32 – -0.08)**	**0.001**	NS	
Number of medications total[a]	**-0.03 (-0.03 – -0.02)**	**<0.001**	**-0.02 (-0.02 – -0.01)**	**<0.001**	**-0.03 (-0.04 – -0.02)**	**<0.001**	**-0.02 (-0.03 – -0.01)**	**<0.001**
Handling own medications	**0.25 (0.18 – 0.32)**	**<0.001**	NS		**0.21 (0.09 – 0.32)**	**<0.001**	NS	
Multidose adherence aid	**-0.26 (-0.34 – -0.18)**	**<0.001**	**-0.10 (-0.19- -0.02)**	**0.014**	**-0.21 (-0.32 – -0.10)**	**<0.001**	**-0-13 (-0.24 – -0.03)**	**0.011**
Hypertension	-0.02 (-0.10 – 0.06)	0.596	NA		0.06 (-0.06 – 0.18)	0.300	NA	
Asthma or COPD	**-0.08 (-0.16 – 0.01)**	**0.072**	NS		**-0.17 (-0.30 – -0.03)**	**0.017**	NS	
Atrial fibrillation	-0.05 (-0.14 – 0.04)	0.276	NA		0.003 (-0.13 – 0.14)	0.965	NA	
Diabetes	**-0.12 (-0.21 – -0.02)**	**0.017**	NS		0.02(-0.14 – 0.17)	0.826	NA	
Heart failure	**-0.08 (-0-20 – 0.03)**	**0.148**	NS		-0.04 (-0.19 – 0.11)	0.600	NA	
Renal failure	0.02 (-0-10 – 0-13)	0.753	NA		-0.06 (-0.20 – 0.08)	0.408	NA	
Anxiety / depression	**-0.14 (-0-28 – -0.01)**	**0.032**	NS		**-0.16 (-0.35 – 0.04)**	**0.117**	NS	
Dementia	-0.11 (-0.33 – 0.11)	0.316	NA		**-0.20 (-0.44 – 0.04)**	**0.103**	NS	
Charlson Comorbidity Index[b]	**-0.03 (-0.05 – -0.02)**	**0.001**	NS		**-0.03 (-0.07 – 0.00)**	**0.090**	NS	

[a] Continuous varable

[b] Tested in a separate model from other comorbidities.

Abbreviations: COPD, Chronic Obstructive Pulmonary Disease; CI, confidence interval; β, regression coefficient; NS, not significant in multivariate regression; NA, not applicable based on univariate regression.

Table S9 Results of univariable and final multivariable mixed model regressions of EQ-5D VAS for the non-long stayers (n=222) and long stayers (n=63)

	Non-long stayers (n=222)				Long stayers (n=222)			
	**Univariable regression coefficient (95% CI)**	**p-value**	**Multivariable regression coefficient (95% CI)**	**p-value**	**Univariable regression coefficient (95% CI)**	**p-value**	**Multivariable regression coefficient (95% CI)**	**p-value**
Constant	56.37 (52.84 – 59.91)	<0.001	103.18 (76.06 – 130.30)	<0.001	53.67 (46.80 **– 60.55**)	<0.001	68.65 (59.41 – 77.89)	<0.001
Intervention group	3.82 (-0.74 – 8.39)	0.101	**4.02 (0.11 – 7.93)**	**0.044**	1.44 (-6.39 – 9.26)	0.719	1.39 (-8.28 – 5.49)	0.692
Time (reference: discharge)								
1 month	3.01 (0.24 – 5.77)	0.033	2.56 (-0.21 – 5.33)	0.070	-3.02 (-9.25 – 3.19)	0.340	-3.70 (-9.89 – 2.50)	0.242
6 months	3.18 (0.33 – 6.03)	0.029	2.58 (-0.26 – 5.43)	0.075	-0.11 (-6.74 – 6.52)	0.973	-0.77 (-7.35 – 5.80)	0.817
12 months	1.87 (-1.12 – 4.86)	0.219	1.07 (-1.93 – 4.06)	0.485	-1.02 (-8.27 – 6.22)	0.782	-1.51-8.69 – 5.68	0.681
Study ward 1	**-9.58 (-14.61 – -4.55)**	**<0.001**	**-7.19 (-11.85 – -2.54)**	**0.002**	**-11.81 (-21.29 – -2.33)**	**0.015**	NS	
Age when included[a]	**-0.67 (-1.03 – -0.30)**	**<0.001**	**-0.38 (-0.72 – -0.03)**	**<0.001**	**-0.52 (-1.12 – 0.07)**	**0.084**	NS	
Sex Female	**-3.50 (-8.16 – 1.16)**	**0.141**	NS		1.63 (-6.13 – 9.39)	0.680	NA	
Level of education >12 years	-0.97 (-5.65 – 3.70)	0.684	NA		**10.12 (2.88 – 17.38)**	**0.006**	NS	
Home-dwelling before included	6.13 (-6.23 – 18.48)	0.331	NA		3.66 (-7.30 – 14.61)	0.513	NA	
Living alone before included	**-3.44 (-8.04 – 1.16)**	**0.143**	NS		-3.71 (-11.23 – 3.82)	0.334	NA	
Home-care services	**-9.78 (-14.19** – -5.37)	**<0.001**	NS		**-11.66 (-18.61 –-4.72)**	**0.001**	**-8.89 (-15.74 – -2.05)**	**0.011**
Number of medications total[a]	**-1.24 (-1.62** – -0.87)	**<0.001**	**-1.16 (-1.52 – -0.81)**	**<0.001**	**-1.11 (-1.76 – -0.46)**	**0.001**	**-0.85 (-1.50 – -0.21)**	**0.010**
Handling own medications	**8.14 (3.63** – 12.66)	**<0.001**	NS		**9.29 (1.94 – 16.65)**	**0.013**	NS	
Multidose adherence aid	**-10.62 (-15.60** – -5.65)	**<0.001**	NS		**-10.90 (-18.49 – -3.31)**	**0.005**	NS	
Hypertension	0.81 (-3.77 – 5.38)	0.730	NA		2.26 (-5.21 – 9.73)	0.554	NA	
Asthma or COPD	-1.75 (-6.69 – 3.18)	0.486	NA		**-6.66 (-14.68 – 1.36)**	**0.104**	NS	
Atrial fibrillation	**-3.85 (-9.01 – 1.32)**	**0.144**	NS		-3.38 (-12.40 – 5.64)	0.463	NA	
Diabetes	**-5.24 (-10.78 – 0.29)**	**0.063**	NS		1.83 (-7.72 – 11.38)	0.707	NA	
Heart failure	**-8.38 (-15.21** – -1.54)	**0.016**	NS		-5.03 (-14.19 – 4.14)	0.282	NA	
Renal failure	-0.34 (-6.84 – 6.16)	0.919	NA		-1.88 (-10.79 – 7.04)	0.680	NA	
Anxiety / depression	**-5.68 (-13.89 – 2.53)**	**0.175**	NS		-3.69 (-16.01 – 8.63)	0.557	NA	
Dementia	0.51 (-15.13 – 16.14)	0.949	NA		-5.54 (-26.33 – 15.24)	0.601	NA	
Charlson Comorbidity Index[b]	**-1.24 (-2.37** – -0.13)	**0.029**	NS		-0.41 (-2.74 – 1.91)	0.728	NA	

[a] Continuous varable

[b] Tested in a separate model from other comorbidities.

Abbreviations: COPD, Chronic Obstructive Pulmonary Disease; CI, confidence interval; β, regression coefficient; NS, not significant in multivariate regression; NA, not applicable based on univariate regression.
